# Single-Molecule Detection Technologies: Advances in Devices, Transduction Mechanisms, and Functional Materials for Real-World Biomedical and Environmental Applications

**DOI:** 10.3390/bios15100696

**Published:** 2025-10-14

**Authors:** Sampa Manoranjan Barman, Arpita Parakh, A. Anny Leema, P. Balakrishnan, Ankita Avthankar, Dhiraj P. Tulaskar, Purshottam J. Assudani, Shon Nemane, Prakash Rewatkar, Madhusudan B. Kulkarni, Manish Bhaiyya

**Affiliations:** 1 Department of Electronics and Communication Engineering, Tulsiramji Gaikwad-Patil College of Engineering and Technology, Nagpur 441108, India; 2 School of Electrical and Electronics Engineering, Ramdeobaba University, Nagpur 440013, India; 3 School of Computer Science and Engineering, Vellore Institute of Technology, Vellore 632014, India; 4 Department of Computer Science and Engineering, Symbiosis Institute of Technology, Nagpur Campus, Symbiosis International (Deemed University), Pune 440035, India; 5 Department of Electronics & Telecommunication Engineering, Shri Sant Gajanan Maharaj College of Engineering, Shegaon 444203, India; dptulaskar@ssgmce.ac.in (D.P.T.);; 6 School of Computer Science and Engineering, Ramdeobaba University, Nagpur 440013, India; 7 Department of Mechanical Engineering, Israel Institute of Technology, Haifa 3200003, Israel; 8 Department of Electronics and Communication Engineering, Manipal Institute of Technology, Manipal Academy of Higher Education (MAHE), Manipal 576104, India; 9 Department of Chemical Engineering and the Russell Berrie Nanotechnology Institute, Technion Israel Institute of Technology, Haifa 3200003, Israel; bhaiyya.manish@gmail.com

**Keywords:** single-molecule detection (SMD), biosensing, diagnostics, surface plasmon resonance (SPR), transistor-based biosensors, optical microfibers, fluorescence-based detection, Raman scattering, recognition tunneling, nanomaterials, sensing, environmental and clinical sensing, point-of-care diagnostics, label-free detection

## Abstract

Single-molecule detection (SMD) has reformed analytical science by enabling the direct observation of individual molecular events, thus overcoming the limitations of ensemble-averaged measurements. This review presents a comprehensive analysis of the principles, devices, and emerging materials that have shaped the current landscape of SMD. We explore a wide range of sensing mechanisms, including surface plasmon resonance, mechanochemical transduction, transistor-based sensing, optical microfiber platforms, fluorescence-based techniques, Raman scattering, and recognition tunneling, which offer distinct advantages in terms of label-free operation, ultrasensitivity, and real-time responsiveness. Each technique is critically examined through representative case studies, revealing how innovations in device architecture and signal amplification strategies have collectively pushed the detection limits into the femtomolar to attomolar range. Beyond the sensing principles, this review highlights the transformative role of advanced nanomaterials such as graphene, carbon nanotubes, quantum dots, MnO_2_ nanosheets, upconversion nanocrystals, and magnetic nanoparticles. These materials enable new transduction pathways and augment the signal strength, specificity, and integration into compact and wearable biosensing platforms. We also detail the multifaceted applications of SMD across biomedical diagnostics, environmental monitoring, food safety, neuroscience, materials science, and quantum technologies, underscoring its relevance to global health, safety, and sustainability. Despite significant progress, the field faces several critical challenges, including signal reproducibility, biocompatibility, fabrication scalability, and data interpretation complexity. To address these barriers, we propose future research directions involving multimodal transduction, AI-assisted signal analytics, surface passivation techniques, and modular system design for field-deployable diagnostics. By providing a cross-disciplinary synthesis of device physics, materials science, and real-world applications, this review offers a comprehensive roadmap for the next generation of SMD technologies, poised to impact both fundamental research and translational healthcare.

## 1. Introduction

Over the past two decades, the ability to observe and analyze single molecules has transitioned from a conceptual challenge into a tangible scientific breakthrough. This capability, known as SMD, is transforming how researchers study biological, chemical, and physical systems. Unlike conventional techniques that examine large groups of molecules and provide averaged data, SMD focuses on the individual behavior of single molecular entities [1,2]. This granularity unlocks insights into the heterogeneity and dynamic nature of molecular processes, information that is often hidden or lost in ensemble measurements. The power of SMD lies in its capacity to capture rare events, stochastic fluctuations, and transient interactions that play critical roles in systems such as enzymatic reactions, protein folding, gene expression, and molecular transport [3,4]. By detecting molecules one at a time, researchers can identify early-stage disease biomarkers, monitor real-time molecular binding kinetics, and reveal subpopulations of molecules that behave differently due to conformational or environmental changes. This level of detail is essential in fields where molecular individuality governs function, such as cancer biology, drug resistance, and neurodegenerative diseases [5,6,7].

**Figure 1:**
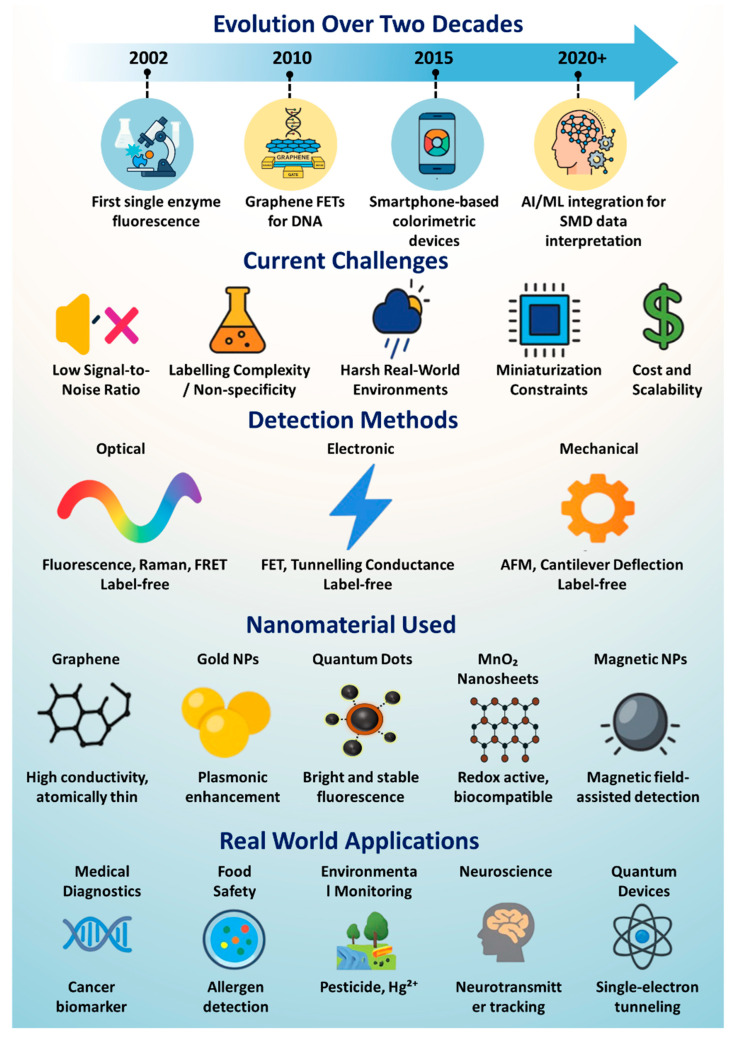
Schematic overview of advancements in SMD, covering its technological evolution, key challenges, detection strategies, enabling nanomaterials, and real-world application domains.

However, detecting a single molecule is no simple feat. Molecules are incredibly small, typically a few nanometers in size, and interact weakly with their surroundings. They often exist in minute concentrations, behave unpredictably in complex environments like blood or soil, and produce signals that are easily drowned out by background noise [8,9]. As a result, SMD systems must achieve extraordinary sensitivity, typically at the femtomolar (10^−15^ M) or attomolar (10^−18^ M) level, while also ensuring specificity, stability, and real-time responsiveness. Achieving this balance requires a combination of innovative detection principles and cutting-edge materials that can transduce molecular events into measurable signals [10]. To meet these challenges, a wide variety of physical and chemical mechanisms have been developed for SMD. These include optical techniques that rely on light–matter interactions (such as fluorescence and Raman scattering), electronic approaches that detect changes in current or voltage (such as transistor-based sensing or tunneling), and mechanical systems that translate molecular binding into force-induced responses [11,12,13,14]. Each method brings unique advantages in terms of speed, resolution, label-free operation, or multiplexing capabilities [15,16,17,18]. The choice of technique often depends on the specific application—whether the goal is to study a protein in solution, detect a contaminant in food, or monitor DNA hybridization on a chip [19]. To contextualize this progress, Figure 1 summarizes the integrated landscape of SMD, highlighting its two-decade evolution, prevailing challenges, detection strategies, enabling nanomaterials, and real-world applications. This schematic serves as a roadmap for understanding the technological trajectory and multidisciplinary scope of the field.

Alongside advances in detection principles, the development of emerging nanomaterials has played a pivotal role in enhancing the performance of SMD platforms [20,21,22]. Materials such as graphene, carbon nanotubes, gold nanoparticles, quantum dots, and magnetic nanostructures bring exceptional electrical, optical, and surface properties that make them ideal for sensing applications. Graphene’s single-atom thickness and outstanding conductivity improve charge-based sensing, while gold nanoparticles enhance optical signals through localized surface plasmon resonance. Quantum dots offer high-brightness and stable fluorescence, making them excellent for long-term single-molecule tracking [23,24,25]. Moreover, 2D nanosheets like MnO_2_ and upconversion nanocrystals are being integrated into sensors to operate in biologically relevant environments without sacrificing performance. These materials not only increase the sensitivity and selectivity but also enable miniaturization, multiplexing, and compatibility with microfluidic and wearable platforms [26].

The value of SMD extends far beyond the laboratory. In the real world, it is emerging as a powerful tool for health, safety, and sustainability. In medical diagnostics, SMD is enabling the detection of cancer biomarkers, viral particles, or proteins in blood or saliva at very early stages, long before symptoms appear [27,28,29,30]. In environmental monitoring, single-molecule sensors are capable of identifying hazardous pollutants like mercury ions, pesticides, or microplastics at trace levels, helping to prevent long-term ecological damage. In food safety, SMD techniques are being used to detect allergens and bacterial contamination in complex food samples, ensuring better consumer protection [31,32,33]. Even in neuroscience and cell biology, these tools are helping scientists to understand the behavior of individual cells and neural pathways in unprecedented detail. Beyond biology, SMD is influencing quantum technologies by detecting single photons or electrons, paving the way for ultrasensitive measurement and imaging systems. Collectively, these applications demonstrate the social and environmental relevance of SMD, empowering fields that directly affect public health, food security, and ecological resilience [34,35,36].

While several excellent reviews have examined various aspects of SMD, most tend to focus on either specific detection methods or narrow application domains [3,7,13,37]. What sets this review apart is its interdisciplinary perspective, which brings together both innovative sensing strategies and material innovations in a unified framework. We highlight how the interplay between device architecture and material properties determines sensor performance, and we critically compare the strengths, limitations, and future potential of different approaches. This integrated view provides researchers, engineers, and technologists with a comprehensive roadmap for advancing SMD across multiple fields, from molecular biology to environmental science and beyond.

## 2. Principles and Devices for Single-Molecule Detection

SMD is an innovative approach that provides unmatched insights into the behavior, interactions, and dynamics of molecules by enabling the observation and analysis of individual molecules. SMD is based on a variety of principles that combine different optical, chemical, and physical processes to provide the sensitivity needed to identify single molecules. This section examines a variety of devices and techniques that have been designed to support SMD; these tools and methods each use distinct mechanisms to accurately detect minute signals from individual molecules. A comparative overview of these techniques is summarized in Table 1, highlighting their typical detection thresholds, transduction modalities, advantages, and limitations across diverse implementation scenarios.

### 2.1. Surface Plasmon Resonance

**Figure 2:**
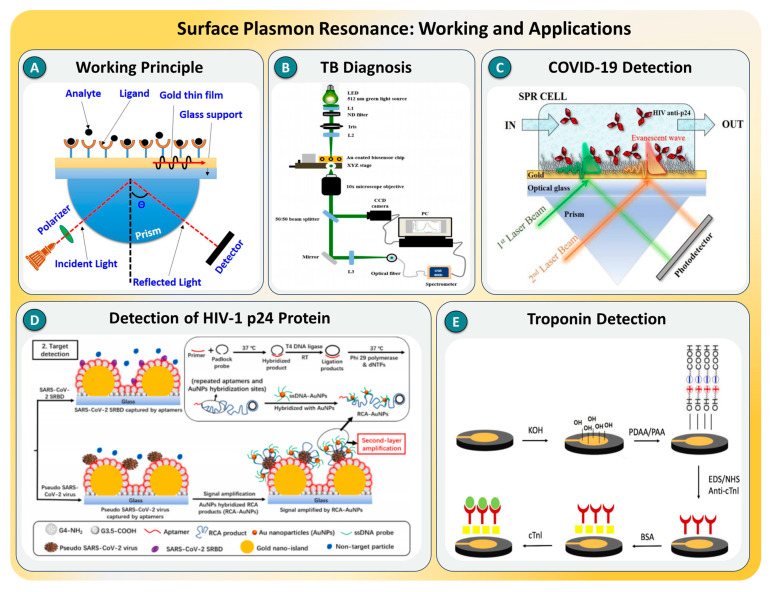
(**A**) Schematic representation of the basic working principle of SPR. (**B**) Experimental setup of an SPR biosensor platform for tuberculosis (TB) diagnosis, taken from [44], with the permission of Elsevier. (**C**) SPR-based aptamer-assisted detection strategy for SARS-CoV-2 using signal amplification, taken from [45], with the permission of Elsevier. (**D**) Dual-laser SPR system for the detection of the HIV-1 p24 protein via evanescent wave interaction, taken from [46], with the permission of MDPI. (**E**) Surface modification strategy for SPR-based detection of cardiac troponin I (cTnI) biomarker, taken from [47], with the permission of Elsevier.

Surface plasmon resonance (SPR) is a powerful optical sensing technique that enables the label-free, real-time detection of molecular interactions by monitoring changes in the refractive index at a metal–dielectric interface, typically gold. When polarized light is directed at this interface under conditions of total internal reflection, it excites collective oscillations of free electrons, known as surface plasmons, on the metal surface, as shown in Figure 2A. This resonance condition is highly sensitive to nanoscale variations in the local refractive index, which occurs when biomolecules bind to functionalized surfaces. The resulting shift in the resonance angle or wavelength provides a quantitative measure of molecular binding. This ability to detect small refractive index changes has enabled SPR to move beyond traditional bulk analysis and into the domain of SMD, especially when enhanced through nanostructures, surface modifications, or amplification strategies [38,39,40,41].

Recent studies have demonstrated the immense potential of SPR in enabling single-molecule sensitivity across a range of biomedical applications. Kochylas et al. [42] developed Si–Ag hybrid plasmonic substrates using single-step chemical etching, achieving a 10^−13^ M detection limit and ∼10^10^ enhancement. This work highlights how engineered plasmonic nanostructures can significantly boost the sensitivity and reproducibility in label-free detection. For example, a hybrid thin-film sensor based on perylene bisimide and nanostructured gold substrates was developed for ultrasensitive dopamine detection, a critical neurotransmitter associated with neurological disorders [43]. A similar principle was applied in tuberculosis diagnostics [44], where the immobilization of mycolic acid antigens on SPR-active gold substrates, followed by enhancement using gold nanoparticles, allowed the sensitive detection of anti-TB antibodies in a label-free format, as shown in Figure 2B. These studies collectively highlight how specific surface chemistry and nanoparticle integration can enable SPR to detect low-abundance targets in real biological samples. The utility of SPR has also been expanded to viral diagnostics, as evidenced by the development of an enhanced LSPR-based platform for detecting SARS-CoV-2 (see Figure 2C) [45]. In this study, the sensor surface was modified with poly(amidoamine) dendrimers that were conjugated to high-affinity aptamers targeting the viral spike protein. The surface modification significantly improved both the ligand density and anti-fouling properties of the chip, achieving a remarkable limit of detection (LOD) of 21.9 pM, over 150-fold more sensitive than conventional antibody-based formats. Similar enhancements were seen in the SPR-based detection of the HIV-1 p24 capsid protein (see Figure 2D) [46], where the covalent immobilization of antibodies via self-assembled monolayers doubled the detection signal compared to physical adsorption, improving the assay’s selectivity and yielding a dissociation constant in the low nanomolar range.

Cardiac biomarker detection using SPR further demonstrates the platform’s translational potential (see Figure 2E). A label-free biosensor functionalized with anti-cardiac troponin I (cTnI) monoclonal antibodies was developed to diagnose myocardial infarctions [47]. The device achieved a detection limit of 0.00012 ng/mL, exhibiting high selectivity and reproducibility in complex biological matrices. An alternative approach employing nanomolecularly imprinted polymers (nanoMIPs) for troponin detection was shown to be effective as a point-of-care SPR sensor [48], delivering excellent binding affinity (K_D = 2.99 × 10^−11^ M) and specificity, even in the presence of structurally similar proteins. These two strategies—antibody-based versus synthetic receptor-based—illustrate the adaptability of SPR to both biological and biomimetic recognition schemes.

In summary, this collection of case studies clearly illustrates the versatility and power of SPR for SMD detection. Through the integration of nanostructures, advanced receptor chemistries, and signal amplification strategies, SPR platforms have evolved to meet the challenges of detecting targets at ultralow concentrations [49,50]. Despite its advantages, several challenges persist, including sensor surface fouling, drift in baseline signals, limited multiplexing, and the need for controlled environments. Addressing these challenges will require further innovation in anti-fouling coatings, miniaturization through microfluidics, and integration with complementary techniques such as electrochemical readouts or machine learning-assisted signal processing. As we advance, the development of robust, low-cost, and portable SPR platforms will be essential to fully realize their potential in clinical diagnostics, neurobiology, environmental monitoring, and personalized medicine. The convergence of SPR with molecular imprinting, aptamer engineering, and data analytics is expected to drive the next generation of ultrasensitive and highly specific SMD technologies [51,52].

### 2.2. Mechanochemical Sensing

**Figure 3:**
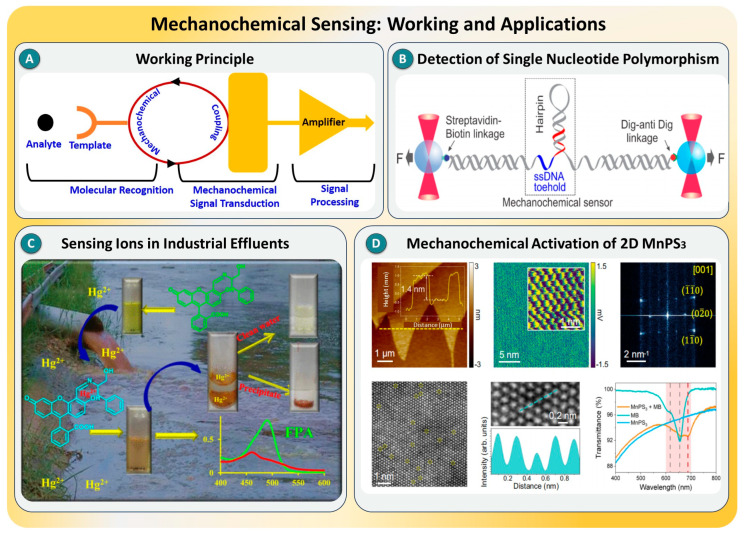
(**A**) Schematic illustrating the working principle of a mechanochemical sensing system involving molecular recognition and signal transduction. (**B**) Mechanochemical sensor design for detecting single-nucleotide polymorphisms using DNA hairpin and force spectroscopy, taken from [53], with the permission of ACS. (**C**) Visual and fluorescence-based mechanochemical detection of Hg^2+^ ions in industrial effluents, taken from [55], with the permission of ACS. (**D**) Structural and spectroscopic characterization of mechanochemically activated 2D MnPS_3_ nanosheets, taken from [56], with the permission of Springer.

Mechanochemical sensing is a rapidly evolving approach in molecular diagnostics that leverages mechanical responses, such as force generation, deformation, or tension modulation, as direct transduction signals triggered by molecular interactions. Unlike conventional optical or electrical detection methods, mechanochemical sensing translates biochemical events into mechanical outputs at the nanoscale, enabling real-time, label-free, and highly sensitive SMD (see Figure 3A) [53,54]. These platforms typically utilize flexible materials like DNA nanostructures or two-dimensional materials, where molecular recognition induces a conformational or mechanical change. This is detected using high-precision tools such as optical tweezers, atomic force microscopy, or plasmon-free surface-enhanced Raman scattering (SERS), creating a direct and specific readout of the molecular interaction.

Recent advances have demonstrated the versatility of mechanochemical platforms across a range of sensing environments. In one study [53], DNA hairpin structures were used as single-molecule mechanochemical sensors capable of detecting antibody–antigen binding (shown in Figure 3B), single-nucleotide mismatches, and microRNAs with femtomolar sensitivity. These DNA “tuning forks” undergo force-mediated transitions that halt upon analyte binding, enabling direct quantification without amplification. Similarly, another study [55] applied this principle to detect physiologically relevant ions like Zn^2+^ and Hg^2+^, with hairpin folding dynamics offering distinct signatures for ion recognition, as shown in Figure 3C. Expanding beyond DNA-based systems, a novel mechanochemical strategy was reported using wrinkled two-dimensional MnPS_3_ nanosheets [56], where strain-induced charge transfer and surface defects enhanced plasmon-free SERS performance, as shown in Figure 3D. The detection of methylene blue down to sub-attomolar (10^−19^ M) levels was achieved by combining mechanical wrinkling with histamine-based functionalization, marking one of the most sensitive nonplasmonic SERS sensors reported to date. Collectively, these studies illustrate how both synthetic and 2D material-based mechanochemical systems are rapidly pushing the limits of single-molecule sensitivity through physical deformation and strain-mediated signal amplification.

Overall, mechanochemical sensing presents a unique transduction paradigm capable of achieving single-molecule resolution without the need for bulky optical labels or complex amplification schemes. Its inherent sensitivity, modularity, and compatibility with a wide range of targets make it a strong candidate for next-generation biosensors. However, current limitations include the need for specialized instrumentation (e.g., AFM or optical traps), the relatively low throughput, and challenges in integrating these sensors into compact, portable formats [57,58]. To address these issues, future work should focus on integrating mechanochemical platforms with microfluidic and electronic systems, exploring new mechanoresponsive materials (e.g., piezoelectric polymers or responsive gels), and combining force-based sensing with orthogonal signal outputs for improved reliability. With continued innovation, mechanochemical sensors hold promise for ultrasensitive detection in fields ranging from precision medicine and infectious disease diagnostics to environmental toxin monitoring [59,60]

### 2.3. Transistor-Based Sensing

**Figure 4:**
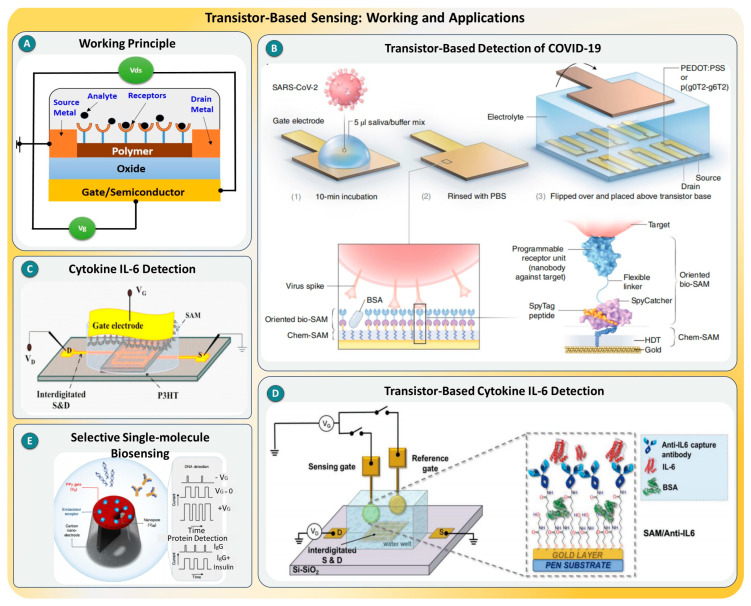
(**A**) Basic working principle of a transistor-based biosensor using gate modulation through analyte–receptor interaction. (**B**) Transistor-based COVID-19 detection using saliva samples and a self-assembled monolayer interface, taken from [63], with the permission of Springer. (**C**) Schematic of IL-6 cytokine detection using a dual-gate transistor sensor with SAM/anti-IL-6-modified gold electrodes, taken from [64], with the permission of Wiley. (**D**) Transistor-based detection of C-reactive protein using interdigitated electrodes and functionalized gate materials, taken from [65], with the permission of Springer. (**E**) Concept of selective single-molecule biosensing using nanopore-transistor integration for DNA/protein identification, taken from [66], with the permission of Springer.

Transistor-based sensing technologies have emerged as powerful tools for SMD, offering label-free, real-time, and miniaturized electronic readouts with exceptional sensitivity. At the heart of these systems are FETs, including electrolyte-gated organic FETs and organic electrochemical transistors, which convert biomolecular interactions at the sensing surface into modulated electrical signals, as shown in Figure 4A. These platforms can be engineered with high-density biorecognition elements, such as antibodies, nanobodies, or aptamers, on the gate electrode or transistor channel, facilitating specific interaction with target analytes. Their electrical amplification capabilities allow even a single molecular event to trigger a measurable current change, making them suitable for rapid diagnostics, particularly in clinical or point-of-care settings [61,62].

Several recent studies have demonstrated the potential of transistor-based sensors in detecting a broad range of biomolecules with single-molecule precision. In a breakthrough approach, Guo et al. [63] developed a nanobody-functionalized OECT capable of detecting SARS-CoV-2 and MERS-CoV antigens at single-molecule levels in unprocessed saliva and nasopharyngeal samples, achieving results in under 15 min, as shown in Figure 4B. Similarly, the SiMoT platform by Scandurra et al. leveraged a large-area EG-FET to detect IL-6 cytokines (see Figure 4C), with an LOD of one molecule per 0.1 mL serum, surpassing standard chemiluminescence-based Simoa arrays [64]. Torsi and colleagues [65] extended this concept to the detection of C-reactive protein (CRP) in saliva using an EGOTFT with a self-assembled monolayer of anti-CRP antibodies, demonstrating ultra-high sensitivity in physiologically relevant fluids, as shown in Figure 4D. Moving forward, Ren et al. introduced nexFET, an integrated nanopore and FET sensor that permits controlled DNA transport and the selective detection of anti-insulin antibodies with enhanced signal-to-noise ratios [66], shown in Figure 4E. Another study demonstrated a transistor-based immunosensor for early cancer biomarker detection, highlighting its potential for multiplexed and miniaturized diagnostics [67]. Moreover, in environmental and clinical biomonitoring, Dibyendu Chowdhury and team reported transistor platforms [68] and showcased their ability to detect hormones and proteins in water samples and biological fluids with excellent selectivity and response times. Collectively, these case studies reveal how transistor-based systems are rapidly moving from proof-of-concept devices toward deployable diagnostic platforms for real-world applications.

In summary, transistor-based biosensors offer a robust and scalable framework for SMD with clear advantages, such as label-free operation, high sensitivity, fast responses, and ease of miniaturization. However, they still face certain limitations, including the impact of Debye screening in physiological media, the challenge of the stable and reproducible functionalization of sensing surfaces, and the integration of complex signal-processing electronics in portable formats. Future work should focus on overcoming signal attenuation in ionic environments, improving gate dielectric engineering, and developing modular, multiplexed systems using low-cost fabrication techniques. Emerging architectures such as 2D material-based FETs, organic semiconductors with volumetric doping, and hybrid bioelectronic–nanopore devices represent exciting directions for next-generation SMD platforms [69,70,71].

### 2.4. Optical Microfibers

Optical microfiber sensors are gaining significant attention for SMD due to their small size, enhanced evanescent fields, and high compatibility with miniaturized platforms. These sensors rely on refractive index modulation at the microfiber–analyte interface, with surface-bound events leading to detectable changes in the phase or intensity of light. However, traditional microfiber sensors often lack sufficient field strength or interface selectivity for SMD. Recent innovations have overcome these limitations by incorporating plasmonic nanostructures, functional coatings, and interferometric coupler geometries, enabling ultrasensitive and label-free biosensing for a wide range of targets [72,73,74].

**Figure 5:**
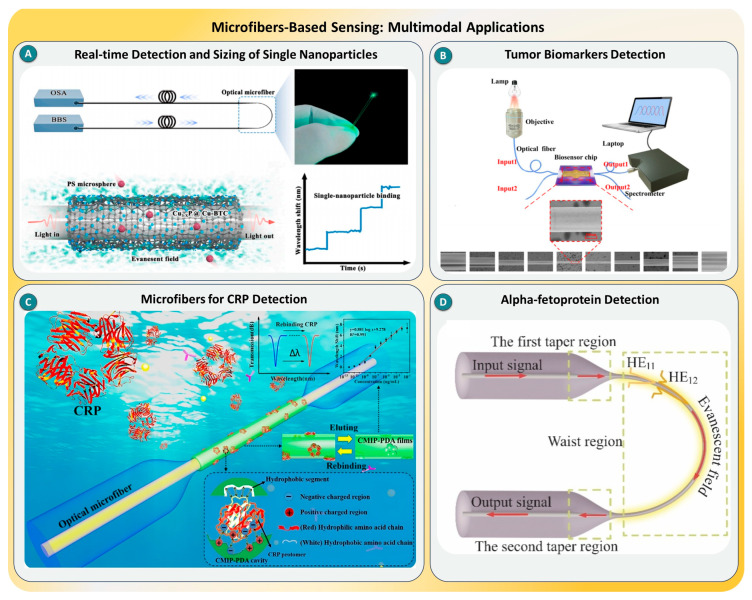
(**A**) Microfiber-based real-time detection and sizing of single nanoparticles via evanescent field interactions, taken from [75], with the permission of Elsevier. (**B**) Optical microfiber biosensor setup for tumor biomarker detection using dual-input/output fiber configuration, taken from [75], with the permission of ACS. (**C**) Schematic of CRP detection using CMIP-PDA-functionalized opt−cal microfiber with specific rebinding regions, taken from [76] with the permission of Elsevier. (**D**) Structural design of a microfiber sensor for alpha-fetoprotein detection using a tapered fiber configuration, taken from [77] with the permission of SSRN.

Several studies have exemplified these advances. A landmark study developed a 3D nanointerface comprising Cu_3−x_P nanocrystals embedded in a Cu-based MOF, engineered to confine and enhance the microfiber’s evanescent field. This design achieved the real-time detection and sizing of individual nanoparticles, highlighting its application for ultrafine environmental particulate monitoring, as shown in Figure 5A [75]. In parallel, a label-free optical microfiber coupler-based biosensor was created to quantify carcinoembryonic antigen in human serum, with a detection limit as low as 34.6 fg/mL (see Figure 5B), while correcting for nonspecific adsorption using Sigma serum [75]. Another sensor based on a polydopamine-mediated molecular imprinting strategy exhibited a detection limit of 5.813 × 10^−10^ ng/mL for CRP (see Figure 5C), attributed to template-induced molecular rearrangements that enhanced the selectivity and binding affinity [76]. Similarly, a U-shaped microfiber coated with Au@WS_2_ nanosheets achieved one of the lowest recorded detection limits, 3.07 × 10^−27^ M, for alpha-fetoprotein (AFP), enabled by synergistic plasmonic enhancement and an increased binding surface area [77], as shown in Figure 5D. These studies, spanning diverse targets from cancer biomarkers to environmental nanoparticles, establish optical microfiber systems as highly tunable and scalable platforms for single-molecule applications.

Together, these studies underscore the vast versatility and performance of optical microfiber systems in single-molecule biosensing. Through innovations in material integration, evanescent field optimization, and structural design, microfiber-based sensors have demonstrated ultralow detection limits across molecular, viral, and particulate targets. Despite their promise, practical implementation is still hindered by fiber fragility, coupling instability, and the complexity of surface modification procedures. To address these challenges, future work should focus on the development of robust protective encapsulation, mass-producible geometries, and intelligent signal processing algorithms. The convergence of photonic engineering, nanomaterials, and microfluidics may soon enable scalable, portable microfiber sensors for real-time clinical diagnostics and environmental applications [78,79,80].

### 2.5. Fluorescence-Based Approaches

**Figure 6:**
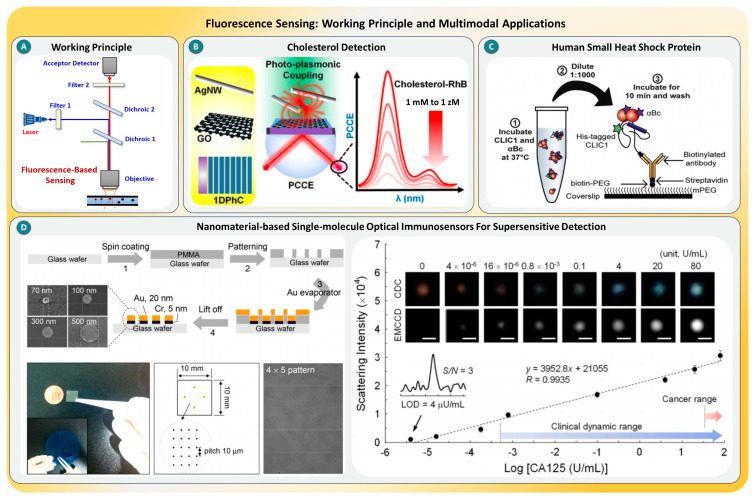
(**A**) Basic working principle of a fluorescence detection system using excitation and emission filters. (**B**) Fluorescence-based cholesterol sensing via photo-plasmonic coupling and emission enhancement, taken from [84] with the permission of ACS. (**C**) Detection of human small heat shock protein using fluorescence−tagged immunoassay with microfluidic incubation, taken from [85] with the permission of Elsevier. (**D**) Nanopatterned optical immunosensor platform for ultrasens−tive SMD of CA125 cancer biomarker, taken from [86] with the permission of Elsevier.

Fluorescence-based SMD is a highly sensitive technique that leverages the light emission from individual fluorophores to identify and study biomolecules at the molecular level. This method works by exciting a fluorophore-labeled molecule with a specific wavelength of light, followed by detecting the emitted fluorescence, as shown in Figure 6A. The spatial and temporal resolution of fluorescence SMD has dramatically improved with innovations such as total internal reflection fluorescence, confocal microscopy, and two-photon excitation [81,82]. These advancements allow the visualization of dynamic processes in real time, making it possible to track the interactions, conformational changes, or enzymatic activity of single biomolecules. Enhancements using nanomaterials, photonic crystals, or plasmonic substrates further amplify signals by manipulating the local electromagnetic environments of fluorophores, often achieving detection limits down to the zeptomolar (10^−21^ M) range [83].

A wide range of studies have showcased the potential of fluorescence-based SMD across diverse platforms and application areas. For instance, a study integrated photonic crystal-coupled emission with graphene oxide and silver nanowires to sense cholesterol at the single-molecule level (see Figure 6B), achieving over a 1300-fold signal enhancement due to synergistic plasmonic interactions [84]. Another study investigated chaperone functionality in human small heat shock proteins using fluorescence-based single-molecule techniques to reveal a two-step interaction mechanism with misfolded proteins, offering critical insights into proteostasis pathways, as shown in Figure 6C [85]. Complementing this, a nanomaterial-integrated optical immunosensor demonstrated the supersensitive detection of antibody–antigen complexes using fluorescence enhancement via plasmonic metals and quantum dots, highlighting their utility in early diagnostics [86], as shown in Figure 6D.

In summary, fluorescence-based SMD offers an exceptional platform for the real-time, ultrasensitive detection of biomolecules, with applications ranging from molecular biology and disease diagnostics to nanomedicine. However, challenges persist, such as fluorophore photobleaching, background fluorescence, and the complexity of signal interpretation in heterogeneous environments. Future advancements should focus on (i) engineering robust fluorophores with high quantum yields and long lifetimes, (ii) integrating machine learning algorithms for single-molecule signal classification, and (iii) developing hybrid platforms combining fluorescence with plasmonic or electrical readouts. Inspired by cutting-edge research, future directions may also include wearable biosensors, in vivo single-cell diagnostics, and multiplexed molecular detection systems that operate in complex biological fluids [87,88].

### 2.6. Raman Scattering

**Figure 7:**
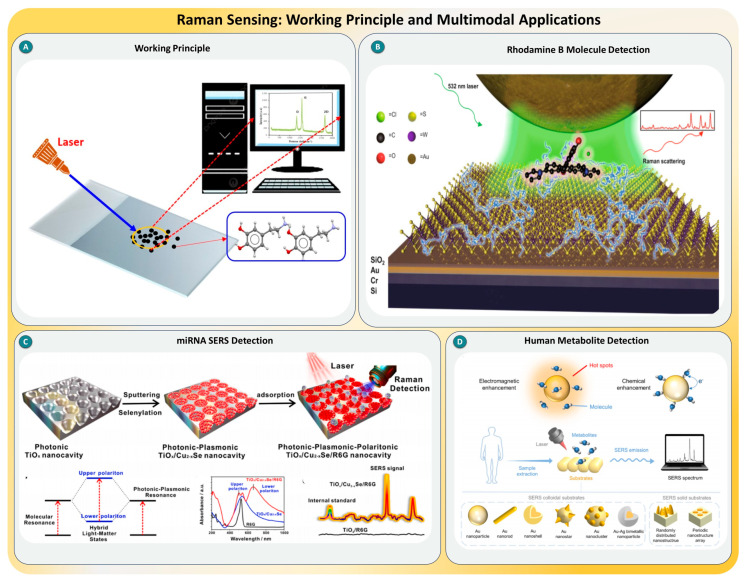
(**A**) Basic working principle of Raman spectroscopy. (**B**) Enhanced Raman signal detection of Rhodamine B molecules using engineered plasmonic substrates, taken from [92] with the permission of Springer. (**C**) Fabrication and utilization of TiO_2_/Cu_2_Se/R6G nanocavity for single-molecule miRNA SERS detection, taken from [94] with the permission of Elsevier. (**D**) Overview of Raman-based detection of human metabolites using various SERS-active substrates for biomedical analysis, taken from [95] with the permission of Elsevier.

Raman scattering is an inelastic light scattering technique that provides a molecular fingerprint by measuring vibrational transitions. For SMD, Raman scattering alone is weak; hence, significant signal enhancement methods are essential. Surface-enhanced Raman scattering leverages metallic nanostructures, typically gold or silver, to amplify Raman signals by up to 10^14^ times through localized surface plasmon resonance [89,90]. Additionally, tip-enhanced Raman scattering and photonic–plasmonic–polaritonic resonators introduce both electromagnetic and chemical enhancement pathways to further boost the sensitivity, allowing the detection of individual biomolecules such as proteins, metabolites, and microRNAs without the need for labels or dyes, as shown in Figure 7A. The integration of nanomaterials and resonant substrates has elevated SERS and TERS into viable platforms for ultrasensitive, label-free, and real-time detection with molecular specificity [91].

Recent research reflects rapid advancement in Raman-based SMD. For instance, Yang et al. demonstrated SMD using WS_2_-Au nanoparticle hybrid structures, achieving enhancement factors up to 10^16^ through synergistic EM and chemical mechanisms, as shown in Figure 7B [92]. Building on this, Bi et al. introduced digital colloid-enhanced Raman spectroscopy, allowing the reproducible quantification of target molecules using single-molecule counting within liquid suspensions [93]. Tian et al. pushed the sensitivity boundaries further by designing photonic–plasmonic–polaritonic nanocavities, achieving detection limits of 1.58 aM for miRNA-21 (see Figure 7D), showcasing hybrid enhancement strategies [94]. Complementarily, Lu et al. reviewed SERS-based metabolite detection in biofluids, detailing its utility for clinical diagnostics and drug monitoring [95]. Across these studies, the trend converges toward combining material engineering, molecular resonance tuning, and statistical signal optimization to refine Raman-based SMD approaches.

In summary, Raman scattering techniques, especially SERS and its derivatives, have proven to be powerful tools for SMD in diverse fields, including biomedical diagnostics, proteomics, metabolomics, and environmental sensing. Despite progress, challenges remain, such as signal variability, hotspot reproducibility, and substrate fabrication. Future directions include integrating Raman systems with AI-driven spectral analysis, developing uniform and reproducible nanostructures, and miniaturizing portable Raman devices for point-of-care diagnostics. The confluence of material science, optics, and molecular biology is poised to make Raman-based SMD a gold standard in next-generation biosensing technologies [96,97,98].

### 2.7. Recognition Tunneling

Recognition tunneling (RT) is a powerful SMD technique that combines molecular recognition with electron tunneling to identify individual analytes. The principle involves a pair of electrodes separated by a nanometer-scale gap, where one or both are functionalized with recognition molecules (e.g., cucurbiturils, imidazole derivatives). When a target molecule bridges this gap, its specific interaction with the capture molecules alters the tunneling current, generating unique, stochastic spike-like patterns [99,100]. These tunneling events are influenced by molecular properties such as hydrogen bonding, the dipole orientation, and the conformational structure, enabling the real-time identification of molecular isomers, analytes, or dynamic states. Since RT operates in aqueous conditions with minimal sample volumes (often <1 picomole), it is highly suited for label-free, electronic single-molecule analysis.

**Figure 8:**
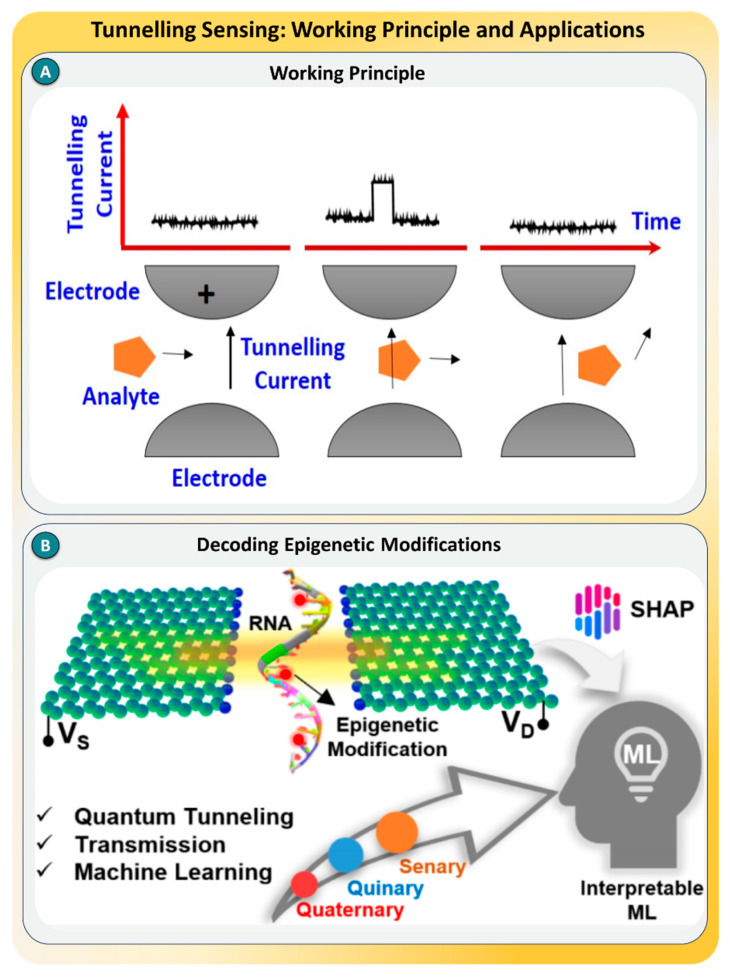
(**A**) Illustration of the tunneling current principle, where analyte passage between electrodes generates measurable electrical signals. (**B**) Direct RNA sequencing using quantum tunneling combined with machine learning for decoding epigenetic modifications, taken from [103] with the permission of ACS.

Recent advances in RT have been demonstrated across diverse applications. Im et al. used RT with imidazole-functionalized electrodes to resolve carbohydrate isomers, such as epimers and anomers, based on distinct tunneling current patterns. This allowed for the electronic classification of stereoisomers that are otherwise indistinguishable by mass spectrometry or ion mobility spectrometry [101]. Similarly, Xiao et al. reported a supramolecular RT approach using cucurbituril (CB7) molecules anchored to both electrodes, enabling host–guest complexation with drug molecules like camptothecin and sanguinarine. Their study showed that the protonation states and pKa values of single drug molecules could be determined electronically via conductance changes in the tunneling signal [102]. Furthermore, recent research has demonstrated the integration of recognition tunneling with data-driven analysis. For example, molecular recognition signals were interpreted using machine learning, such as support vector machines (SVMs), enabling classification accuracies of over 99% in distinguishing molecular features based on hundreds of extracted signal features, as shown in Figure 8B [103].

In summary, recognition tunneling has emerged as a label-free, ultrasensitive, and information-rich technique for identifying small molecules, structural isomers, and biomolecular conformers at the single-molecule level. The major strengths of RT lie in its ability to resolve analytes in complex mixtures, its ultralow sample requirements, and its potential for electronic integration into portable diagnostics. However, challenges remain, such as ensuring reproducibility across electrodes, minimizing noise from the surrounding environment, and standardizing signal interpretation. Future directions involve coupling RT with nanopore sequencing platforms, applying advanced AI algorithms for real-time signal classification, and expanding the library of recognition chemistries for broader target specificity. With continued development, RT could revolutionize real-time single-molecule analytics in fields ranging from glycomics and drug discovery to personalized diagnostics [104,105].

### 2.8. Nanopore-Based Sensing

Nanopore sensing has become a cornerstone technique for single-molecule analysis, providing direct electrical readouts of individual biomolecules as they translocate through nanometer-scale pores under an applied potential. Each transient current blockade corresponds to the molecule’s size, charge, and conformation, enabling the label-free identification of nucleic acids, peptides, proteins, and metabolites [106,107,108]. Biological nanopores (e.g., α-hemolysin, MspA) offer intrinsic molecular selectivity, whereas solid-state nanopores (e.g., SiN_x_, graphene, MoS_2_) provide mechanical robustness, chemical stability, and tunable geometries. Recent advances in hybrid nanopores and surface functionalization have improved the signal stability and molecular discrimination, broadening their use in DNA sequencing, protein fingerprinting, and pathogen or biomarker detection. Importantly, several functional materials discussed in Section 3, namely graphene, carbon nanotubes, and metallic nanoparticles, are being increasingly integrated into nanopore architectures to enhance the conductivity and transduction efficiency. Beyond nucleic acid sequencing, nanopore platforms are now being applied to cancer diagnostics, virus characterization, and multi-omic sensing. Recent works further highlight this evolution.

**Figure 9:**
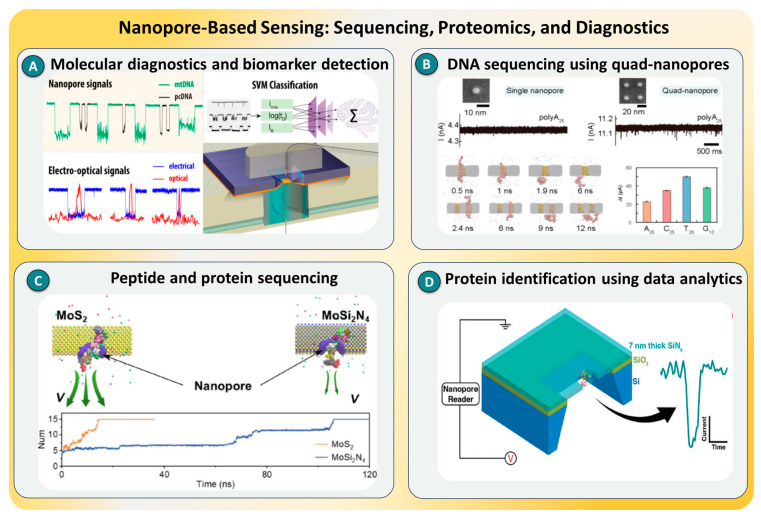
Representative applications of nanopore-based SMD platforms. (**A**) Molecular diagnostics and biomarker detection using solid-state nanopores with electro-optical and ML readouts, taken from [109] with the permission of ACS. (**B**) DNA sequencing via quad-nanopore arrays, enabling improved base discrimination, taken from [110] with the permission of ACS. (**C**) Peptide and protein sequencing using MoSi_2_N_4_ nanopores for slowed translocation and higher resolution, taken from [111] with the permission of ACS. (**D**) Protein identification through solid-state nanopores integrated with data analytics frameworks, taken from [112] with the permission of Wiley.

Pal et al. [109] developed an amplification-free solid-state nanopore assay for mitochondrial DNA quantification directly from cancer and blood samples, achieving >95% classification accuracy without PCR bias, as illustrated in Figure 9A. Hu, Rui et al. [110] introduced a quad-nanopore array, shown in Figure 9B, that slowed translocation about fivefold and discriminated DNA homopolymers with a single-nucleotide resolution at low bias. Zhang et al. [111] employed MoSi_2_N_4_ nanopores to regulate peptide mobility for high-fidelity protein sequencing, as represented in Figure 9C, while Dutt et al. [112] integrated solid-state nanopores with advanced pattern recognition algorithms for accurate protein identification (Figure 9D). In addition, Motone et al. [113] achieved the multi-pass reading of intact protein strands using a motor-assisted nanopore system, marking a major step toward complete protein sequencing. These works discuss innovations in materials, architecture, and data analytics that are propelling nanopore-based SMD toward comprehensive, high-precision molecular diagnostics.

**Table 1:** Comparison of single-molecule detection techniques.

SMD Technique	Label-Free	Typical Detection Limit	Transduction Type	Advantages	Limitations	Ref.
Surface Plasmon Resonance (SPR)	Yes	Attomolar (10^−18^ M)	Optical (Refractive Index)	Real-time, label-free, high specificity	Surface fouling, baseline drift	[114,115]
Mechanochemical Sensing	Yes	Femtomolar to Attomolar	Mechanical/Force-Based	Direct force measurement, high sensitivity	Low throughput, complex setup	[53,116]
Transistor-Based Sensing	Yes	Single Molecule	Electrical	Miniaturized, electronic readout, real-time	Debye screening, surface instability	[117,118]
Optical Microfiber	Yes	Zeptomolar (10^−21^ M)	Optical (Phase/Intensity Shift)	Compact, scalable, ultrasensitive	Fiber fragility, complex modification	[119,120]
Fluorescence-Based Detection	No	Zeptomolar (10^−21^ M)	Optical (Fluorescence)	High spatial/temporal resolution, multiplexing	Photobleaching, autofluorescence	[121]
Raman Scattering (SERS/TERS)	Yes	Single Molecule	Optical (Inelastic Scattering)	Molecular fingerprinting, label-free	Hotspot variability, substrate fabrication	[122]
Recognition Tunneling	Yes	Single Molecule	Electronic (Tunneling Current)	Molecular specificity, ultralow volume	Reproducibility, signal variability	[102]

While advancements in detection principles have significantly expanded the scope and performance of SMD platforms, their ultimate sensitivity, selectivity, and real-world deployability are often constrained by fundamental challenges in signal transduction, background suppression, and device miniaturization. These limitations have driven a parallel surge in the development of novel functional materials engineered specifically to augment or even redefine traditional sensing mechanisms. Materials such as carbon nanostructures, quantum dots, plasmonic nanoparticles, and catalytic 2D sheets have not only enhanced signal amplification and molecular recognition but also enabled new modes of detection through tailored optical, electrical, magnetic, and catalytic interactions. The following section systematically explores how these emerging materials have become integral to next-generation SMD technologies, serving not merely as substrates or labels but as active, multifunctional components that fundamentally reshape the capabilities and design of single-molecule sensors.

## 3. Emerging Materials for Single-Molecule Detection

The progression of SMD technologies has been fundamentally driven by advances in material engineering, which directly influence the transduction efficiency, resolution, and operational robustness of detection platforms. As the field advances toward femtomolar and attomolar detection thresholds, the choice of materials becomes paramount in governing critical performance metrics such as molecular recognition specificity, signal amplification, the signal-to-noise ratio (SNR), and compatibility with diverse transduction modalities. Beyond serving as structural substrates, these materials function as active interfaces that convert subtle molecular events into quantifiable signals with high fidelity and often in real-time and under physiologically or environmentally complex conditions [23].

This section systematically explores six major classes of emerging materials that have demonstrated transformative potential in SMD: (1) graphene and carbon nanotubes, which provide exceptional electrical conductivity and surface areas for label-free electronic sensing; (2) quantum dots, offering tunable, high-brightness emission with minimal photobleaching for optical tracking; (3) metallic and polymeric nanoparticles, enabling plasmonic and fluorescent signal enhancement across multiplexed platforms; (4) upconversion nanocrystals, facilitating deep-tissue, autofluorescence-free detection under near-infrared excitation; (5) MnO_2_ nanosheets, contributing redox-active and catalytic functionalities for electrochemical signal generation; and (6) magnetic nanoparticles, which allow for magnetic enrichment, dynamic manipulation, and contactless readout. These materials are foundational in enhancing SMD capabilities across a wide spectrum of applications, including molecular diagnostics, intracellular imaging, point-of-care biosensing, and environmental surveillance.

**Figure 10:**
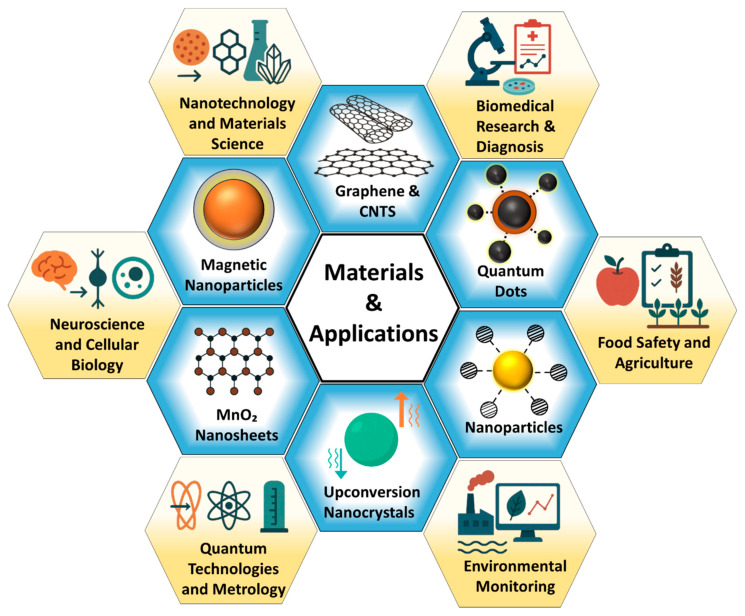
Schematic illustrating key materials (e.g., MNPs, graphene, QDs, MnO_2_, UCNPs) and their major application domains in SMD.

A schematic overview of these six material classes and their principal application domains is provided in Figure 10, illustrating their distinct roles and integration within state-of-the-art SMD architectures. Each material category will be discussed in detail in the following subsections, supported by representative case studies. These discussions will critically evaluate their sensing mechanisms, performance benchmarks, and integration potential with conventional and next-generation detection systems, while also identifying existing limitations and outlining future directions for material-driven innovations in SMD.

### 3.1. Graphene and Carbon Nanotubes (CNTs)

Carbon nanomaterials, particularly carbon nanotubes (CNTs) and graphene, have emerged as pivotal components in advancing SMD technologies. Their atomic thickness, remarkable electrical conductivity, and extensive surface area offer unique transduction capabilities at the molecular scale [123,124]. CNTs, characterized by quasi-one-dimensional structures and ballistic electron transport, enable real-time electrical readouts with minimal noise interference. Similarly, graphene, the prototypical two-dimensional material, presents extraordinary mechanical strength, near-uniform optical absorption, high carrier mobility, and quantum-scale electronic features. Both materials exhibit inherent label-free sensing potential and compatibility with nanoscale device fabrication, positioning them as ideal transduction scaffolds in SMD platforms. Their π–π stacking interactions and high surface-to-volume ratios further facilitate specific molecular interactions, thereby enhancing the detection specificity and reducing background interference [125].

**Figure 11:**
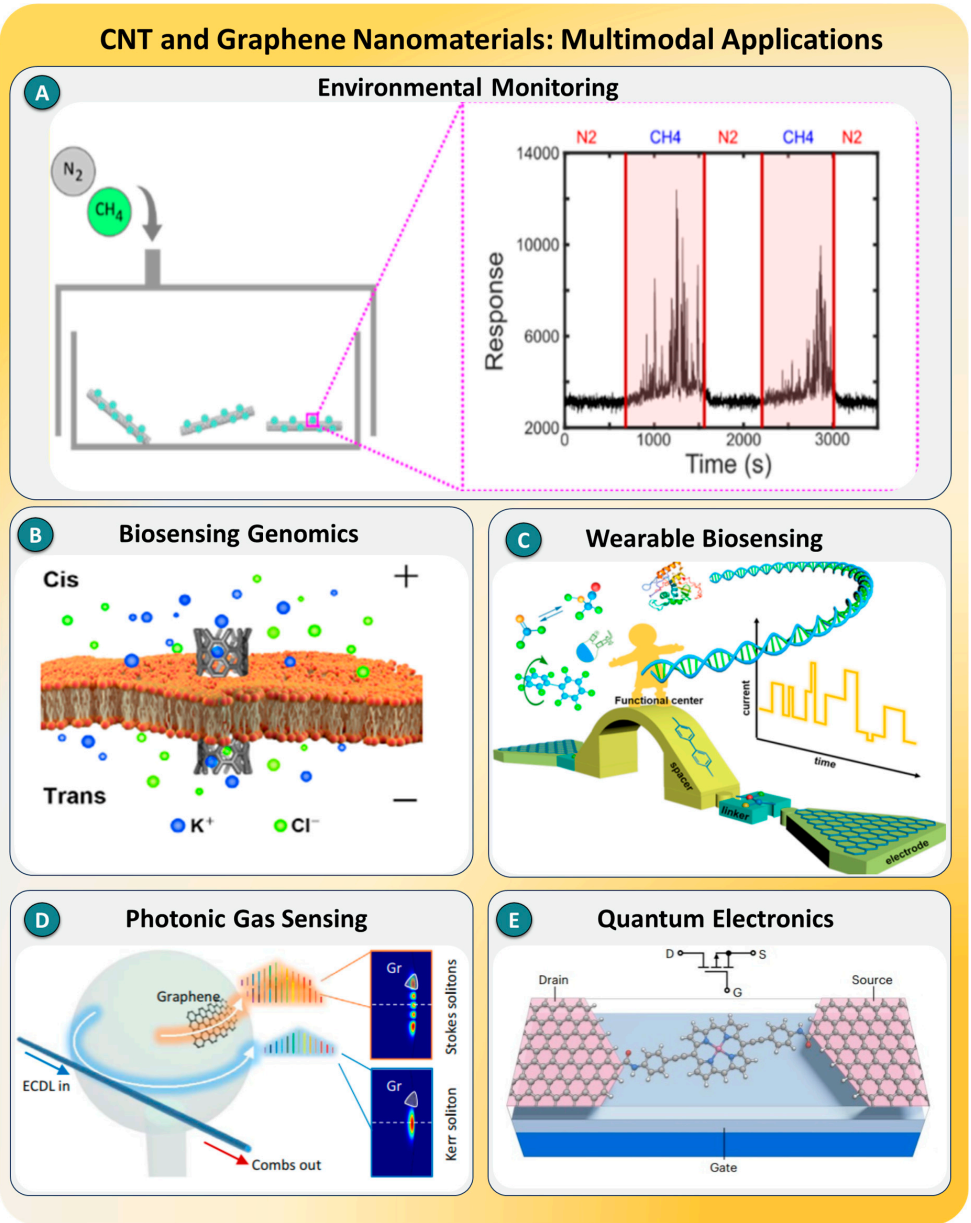
Representative case studies demonstrating the role of carbon nanomaterials in SMD. CNT-based platforms include (**A**) biomimetic ion transport channels for genomic sensing, reprinted with permission from Ref. [128], *Nature*; (**B**) ultralow-concentration methane detection for environmental monitoring, reprinted with permission from Ref. [129], ACS. Graphene-based systems include (**C**) wearable biosensors for real-time nucleic acid detection, reprinted with permission from Ref. [132], ACS; (**D**) photonic gas sensing via soliton generation in microresonators, reprinted with permission from Ref. [133], *Nature*; (**E**) Quantum interference-enabled molecular transistors for precision electronics, reprinted with permission from Ref. [134], ACS.

Recent advances in CNT-based platforms have significantly enhanced the performance of SMD systems, transitioning from static electronic junctions to dynamic, real-time biosensors. M. P. Landry et al. [126] demonstrated graphene single-molecule junctions, which were fabricated using lithographic nanogaps, enabling the picoampere-level electrical detection of noncovalent interactions with a sub-nanometer resolution. Building on this, Refs. [127,128] introduced defect-engineered CNT architectures with improved anchoring and dual-channel modes, facilitating the real-time tracking of nucleic acid hybridization events at detection limits below 10 fM and signal-to-noise ratios exceeding 25 dB (Figure 11A). In 2023, X. Gong et al. [129] introduced palladium-functionalized, near-infrared fluorescent CNTs, which enabled methane sensing at 0.7 ppb with a <1 s response time, illustrating the extension of CNT sensors beyond biomolecular targets, as shown in Figure 11B. Most recently, Y. Lee et al. [130] reported serotonin-specific smFETs based on CNTs that resolved aptamer conformational changes with a microsecond resolution and nanomolar dissociation constants.

Graphene-based nanostructures have rapidly gained prominence in SMD due to their exceptional electrical conductivity, biocompatibility, high carrier mobility, and tuneable surface chemistry. In 2021, I. Kamińska et al. [131] reported a graphene oxide field-effect transistor (GO-FET) functionalized with DNA aptamers, which was shown to detect dopamine down to 1 fM, leveraging π–π interactions and electrostatic gating for selectivity and label-free detection. Expanding into wearable biosensing, Ref. [132] integrated reduced graphene oxide (rGO) with textile-based microfluidics, achieving real-time cortisol detection with a sub-minute response time and high stability in sweat matrices (Figure 11C). Meanwhile, T. Tan et al. [133] demonstrated laser-engraved 3D graphene electrodes combined with ionic liquid gating for ultrasensitive NO_2_ gas detection (LOD ~12 ppb), highlighting the adaptability of graphene structures across analyte types, as illustrated in Figure 11D. A major advancement was introduced by S. Ganesh’s group [134], where a 3D graphene oxide quantum sensor (GOQS) achieved single-molecule sensitivity (LOD ~10^−15^ M) with an enhancement factor of 10^14^ via quantum confinement and edge effects, outperforming many plasmonic SERS platforms (Figure 11E). Finally, Z. Chen et al. [135] exploited quantum interference in zinc porphyrin/graphene molecular transistors, demonstrating over 10^4^ conductance-switching ratios and a subthreshold swing at the thermionic limit, enabled by destructive quantum interference between graphene edge states and molecular orbitals.

As illustrated in Figure 11, the reviewed case studies highlight the diverse implementations of CNT- and graphene-based architectures in SMD, demonstrating their unique capabilities across biochemical and environmental applications. These platforms enable label-free, real-time sensing with femtomolar to attomolar sensitivity and microsecond-scale temporal resolutions, well beyond the reach of conventional ensemble-based techniques. CNTs offer quasi-one-dimensional conductivity, structural tunability, and aptamer integration for the tracking of dynamic molecular events, while graphene enables quantum interference-based gating, Raman enhancement, and wearable-format sensing [136]. Despite these advantages, both materials face limitations, such as fabrication inconsistencies, ambient instability, and surface fouling. Emerging strategies such as defect engineering, nanohybrid design, and AI-enhanced signal analysis are being explored to overcome these constraints. Looking forward, the convergence of carbon nanomaterials with quantum electronics, flexible substrates, and machine learning is poised to shape the next generation of ultrasensitive, portable SMD technologies [137,138].

### 3.2. Quantum Dots

Quantum dots (QDs) are nanoscale semiconductor crystals that exhibit size-tunable optical and electronic properties due to quantum confinement effects. Their discrete energy levels enable narrow, high-brightness emission spectra, while their high photostability and large Stokes shifts make them particularly well suited for prolonged and multiplexed imaging applications. Unlike traditional organic dyes and fluorescent proteins, QDs resist photobleaching and blinking when surface-engineered appropriately, allowing for the continuous tracking of single molecules over extended periods [139,140]. Moreover, their broad absorption and sharp emission profiles support the simultaneous excitation of multiple colors, making them ideal candidates for high-throughput, multi-target biosensing. In SMD, QDs serve not only as robust fluorescent reporters but also as components in energy transfer systems, super-resolution imaging, and photothermal sensing. Their unique optical properties, combined with their surface functionalizability, allow precise targeting, low-background signal generation, and integration into microfluidic, electrochemical, or nanophotonic platforms. These attributes have positioned QDs as one of the most powerful classes of materials for real-time, in vitro, and in vivo SMD applications, ranging from nucleic acid diagnostics to protein conformational studies and intracellular dynamics [141].

**Figure 12:**
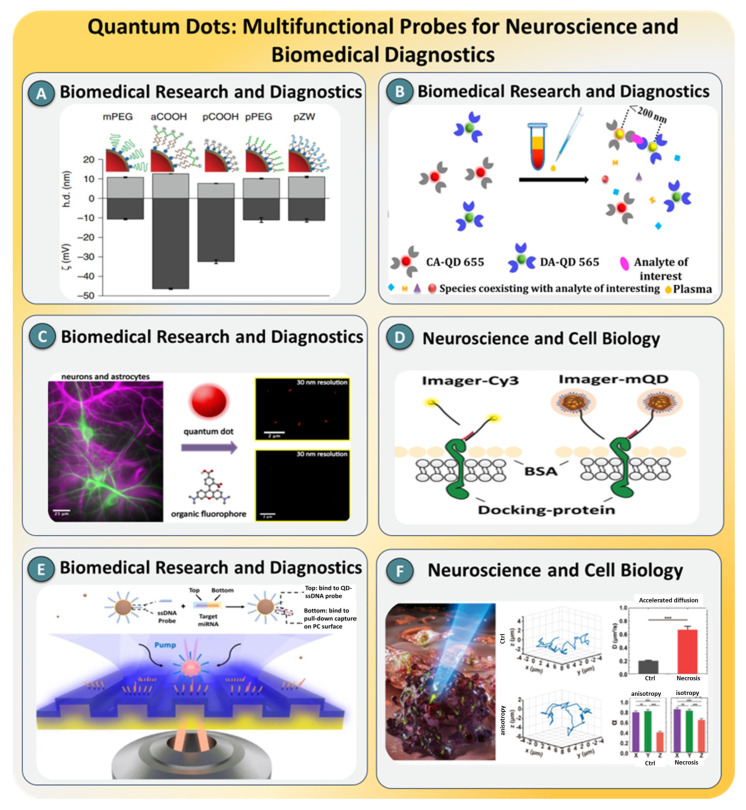
Representative case studies demonstrating the versatility of QD-based platforms for SMD. (**A**) Surface charge and hydrodynamic diameter variation across PEG-modified QDs for optimized biomedical targeting, reprinted with permission from Ref. [142], *Nature*. (**B**) Dual−color QD assay for detection of analytes in plasma amid coexisting species with high specificity, reprinted with permission from Ref. [144], ACS. (**C**) Multicolor QDs enabling high-resolution imaging of ne−rons and astrocytes with superior spatial clarity over organic dyes, reprinted with permission from Ref. [145], ACS. (**D**) QD-based single-particle tracking for mapping of membrane–protein interactions in live cells, reprinted with permission from Ref. [146], *Nature*. (**E**) QD-mediated microfluidic chip for miRNA detection using dual-probe hybridization and optical pumping, reprinted with permission from Ref. [147], *Nature*. (**F**) QD-assisted diffusion analysis for probing of cellular necrosis, highlighting changes in intracellular transport behavior, reprinted with permission from Ref. [148], ACS.

Recent advances in QD-based SMD platforms have significantly expanded the scope of ultrasensitive and multiplexed molecular analysis. M. U. Zahid’s group [142] proposed CdSe/ZnS QDs conjugated with thrombin-specific aptamers, which enabled femtomolar-level detection through Forster resonance energy transfer, offering precise signal discrimination with minimal background interference, as shown in Figure 12A. Expanding the multiplexing capabilities, K. A. Knapper et al. [143] employed dual-emission QDs coupled with upconversion nanoparticles to simultaneously track microRNA and protein biomarkers within single cells, achieving picomolar sensitivity under physiological conditions. In addition, K. A. Knapper et al. [144] integrated QDs into a DNA walker-based ratiometric fluorescent platform, which enabled nucleic acid detection with zeptomolar-level sensitivity, exploiting catalytic hairpin assembly for amplified signal outputs (Figure 12B). Transitioning toward digital bioassays, J. M. Urban’s group [145] demonstrated a QD-encoded immunoassay on a digital microfluidic chip, as illustrated in Figure 12C, achieving detection limits below 100 aM with a total assay time of under 30 min. Super-resolution imaging was advanced by Y. Chang et al. [146] using DNA-PAINT QDs, which circumvented blinking artifacts and achieved enhanced spatial precision, surpassing conventional fluorophore-labeled probes, as presented in Figure 12D. In 2020, Y. Xiong et al. [147] combined QD-conjugated nanobodies with SPAD arrays to detect single biomarkers with <10 pM sensitivity, even in high-noise cellular environments (Figure 12E). More recently, H. Y. Luo and team [148] used QD-based three-dimensional single-particle tracking to map subcellular diffusion in necrotic cells, as shown in Figure 12F, revealing anisotropic-to-isotropic transition patterns associated with cytoskeletal degradation. Likewise, S. Hao [149] introduced a photothermal detection strategy using QDs and whispering gallery mode resonators, enabling the label-free detection of 5 nm QDs with heat dissipation thresholds as low as 0.75 pW and signal-to-noise ratios exceeding 10^4^. Collectively, these case studies illustrate the remarkable versatility of QDs in enabling high-resolution, ultrasensitive, and real-time SMD across both biological and synthetic systems.

As illustrated in Figure 12, the surveyed case studies underscore the growing utility of QDs in expanding the functional and analytical capabilities of SMD platforms. These include breakthroughs in signal enhancement via nanostructured photonic surfaces, ultrastable intracellular tracking with nanometer-scale spatial precision, and high-contrast photothermal sensing. Compared to conventional fluorophores, QDs offer unmatched brightness, photostability, and multiplexing potential, enabling the prolonged observation of single-molecule events and complex molecular interactions. Nevertheless, limitations such as cytotoxicity, nonspecific binding, and fluorescence intermittency (blinking) persist, particularly in biological environments [150]. Surface passivation strategies, such as polymer coatings or shell thickening, as well as hybridization with photonic crystals and plasmonic nanostructures, are actively being explored to overcome these issues. Looking ahead, the integration of QDs with AI-based trajectory analysis, real-time tracking in deep tissues using NIR-II emission, and biocompatible QD formulations for clinical diagnostics are expected to drive the next phase of QD-enabled single-molecule technologies [151,152].

### 3.3. Nanoparticles

Metallic and polymeric nanoparticles have emerged as powerful platforms in SMD owing to their tunable physicochemical properties and capacity for intense signal amplification. Noble metal nanoparticles, particularly gold and silver, exhibit strong localized surface plasmon resonance (LSPR), which concentrates electromagnetic fields at the nanoscale to significantly enhance optical signals via mechanisms such as surface-enhanced Raman scattering (SERS) [153]. These plasmonic features, combined with large surface-area-to-volume ratios, enable functionalization with molecular probes for highly specific recognition and transduction. In parallel, polymeric and hybrid nanoparticles contribute superior biocompatibility, colloidal stability, and surface passivation, expanding the operational robustness of nanoparticle-based sensors. Together, these classes of nanostructures have been applied across diverse SMD modalities, including SERS, single-particle tracking (SPT), photothermal sensing, and near-field microscopy, to achieve ultrahigh spatial and temporal resolutions at attomolar detection limits [154,155].

**Figure 13:**
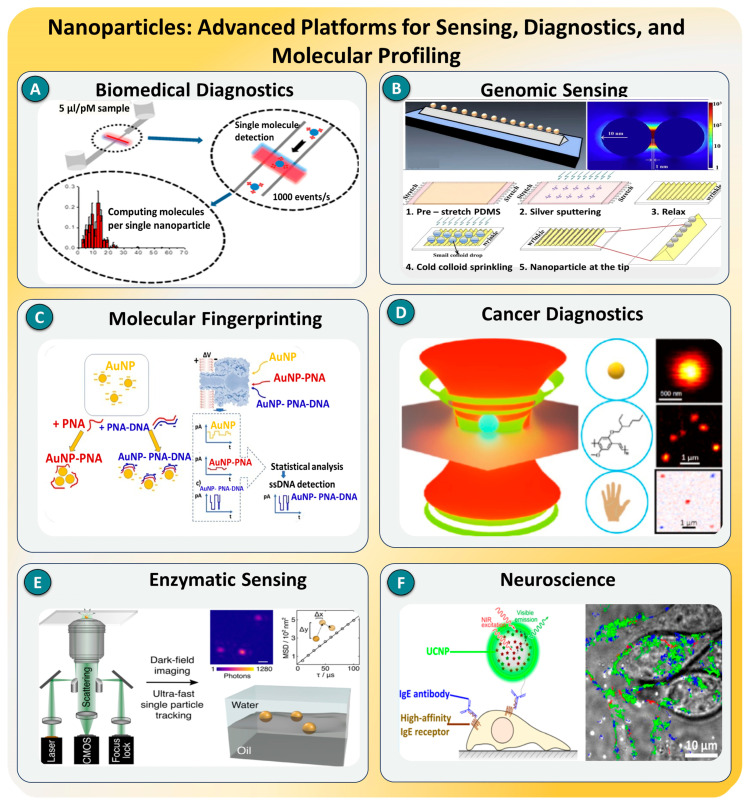
Overview of nanoparticle-enabled SMD approaches across diverse biomedical applications. (**A**) Plasmon-enhanced digital counting of single-molecule events per nanoparticle for biomedical diagnostics, reprinted with permission from Ref. [156], ACS. (**B**) Gold nanoparticle arrays structured via stretchable substrates for high-sensitivity genomic sensing, reprinted with permission from Ref. [157], Elsevier. (**C**) Gold nanoparticle–peptide nucleic acid (AuNP–PNA) complexes for electrochemical molecular fingerprinting and single-stranded DNA detection, reprinted with permission from Ref. [158], *Nature*. (**D**) Optical trapping and detection of antibody-functionalized nanoparticles for cancer biomarker identification, reprinted with permission from Ref. [12], ACS. (**E**) Single-particle tracking using gold nanoparticles and dark-field imaging for enzyme activity monitoring, reprinted with permission from Ref. [160], ACS. (**F**) Upconversion nanoparticles for visualization of IgE receptor-labeled immune cells in live tissues, supporting applications in neuroscience, reprinted with permission from Ref. [161], ACS.

Recent advancements in plasmonic and hybrid nanomaterials have significantly broadened the sensitivity and multiplexing capacity of SMD. S. R. Jung et al. [156] demonstrated a silver nanoparticle–graphene oxide (AgNP–GO) hybrid platform for enhanced SPR-based biosensing. This platform is illustrated in Figure 13A, achieving the femtomolar detection of cardiac troponin I with excellent reproducibility and minimal nonspecific adsorption. Then, H. Guo [157] built upon this by fabricating highly ordered gold nanoparticle arrays with nanogaps below 10 nm (Figure 13B), yielding surface-enhanced Raman scattering (SERS) enhancement factors up to 10^7^ and enabling the dynamic monitoring of DNA structural shifts. In 2020, L. Mereuta et al. [158] reported dielectric–metal hybrid nanorods incorporated into whispering gallery mode resonators, which demonstrated enhanced local field confinement and reached Raman detection limits as low as 10^−21^ M for small-molecule analytes, as demonstrated in Figure 13C. S. Adhikari et al. [12] designed a nanoplasmonic biosensor based on nano-island gold films, as shown in Figure 13D, that enabled the simultaneous detection of multiple cancer markers (e.g., PSA and CEA) with LODs in the range of 1–5 fM, highlighting multiplexing’s potential for early-stage diagnostics.

Incorporating magnetic functionality, K. Trofymchuk et al. [159] presented dual-mode magnetic–plasmonic nanoparticles for miRNA sensing, with an LOD of 50 aM, combining magnetic enrichment and optical amplification in serum matrices. A novel catalytic amplification route was introduced by X. Meng et al. and team [160], as shown in Figure 13E, through Au@Cu_2_O core–shell nanocubes that exhibited peroxidase-like activity, achieving the colorimetric detection of hydrogen peroxide at 10^−6^ M and enabling SMD via a dual-readout approach. Recently, O. Dukhno et al. [161] demonstrated live-cell-compatible SMD using upconversion nanoparticles conjugated to IgE antibodies, enabling the real-time tracking of FcεRI receptor diffusion in mast cells with a single-particle resolution and no background fluorescence, thanks to their anti-Stokes emission and photobleaching resistance, as shown in Figure 13F.

As illustrated in Figure 13, these studies collectively demonstrate the multifaceted utility of nanoparticle-based systems in SMD, spanning high-contrast optical detection, single-molecule tracking, and photonic signal amplification. Compared to traditional labels, nanoparticles offer superior brightness, photostability, and multiplexing capabilities. However, challenges such as particle aggregation, surface fouling, and batch-to-batch variability in functionalization persist. Current strategies to address these issues include silica shelling, ligand exchange chemistry, polymer encapsulation, and precision nanofabrication. Looking forward, the fusion of nanoparticle-enhanced SMD platforms with AI-guided analytics, quantum plasmonic systems, and flexible integration into lab-on-a-chip devices is expected to drive new frontiers in ultrasensitive, field-deployable molecular diagnostics [162,163].

### 3.4. Upconversion Nanocrystals

Upconversion nanocrystals (UCNCs), typically composed of rare earth-doped fluoride matrices, have emerged as highly promising optical transducers in SMD due to their ability to convert low-energy near-infrared (NIR) excitation into higher-energy visible emission via anti-Stokes processes. This upconversion luminescence (UCL) confers several intrinsic advantages over conventional downconversion fluorophores, including minimized autofluorescence, reduced photobleaching, and deep-tissue penetration—critical attributes for high-fidelity biosensing in complex biological matrices [164,165]. Furthermore, UCNCs exhibit long luminescence lifetimes, narrow emission bands, and excellent photostability, enabling multiplexed, time-gated, and ratiometric detection schemes with high signal-to-noise ratios. Recent advancements in core–shell architectures, lanthanide doping strategies, and surface modification have significantly enhanced their quantum efficiency, spectral tunability, and biointerfacing, broadening their applicability across both in vitro diagnostics and in vivo imaging platforms [166].

**Figure 14:**
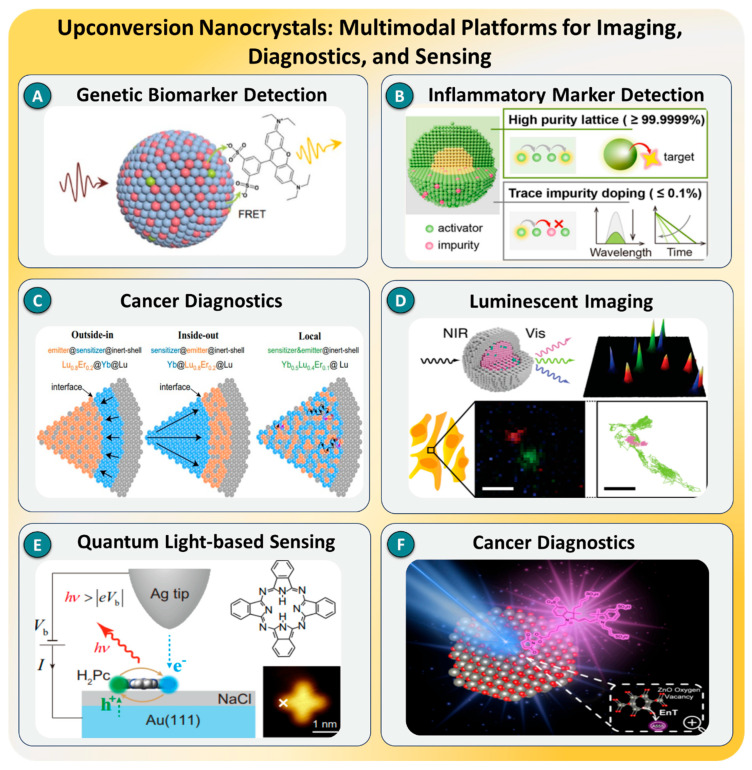
This figure presents distinct implementations of upconversion nanocrystals (UCNPs) for advanced molecular diagnostics. (**A**) Multishell UCNPs are designed for ratiometric genetic b−omarker sensing through efficient energy transfer, reprinted with permission from Ref. [167], Elsevier. (**B**) High-purity lattices and precise impurity doping allow enhanced emission fidelity for inflammatory marker detection, reprinted with permission from Ref. [167], Elsevier. (**C**) Structural engineering approaches—outside in, inside out, and local—optimize the diagnostic signal strength and multiplexing for cancer-related targets, reprinted with permission from Ref. [168], Springer. (**D**) UCNPs are applied in high-contrast, multiplexed luminescent imaging under biological conditions, reprinted with permission from Ref. [169], ACS. (**E**) UCNP-based quantum electroluminescent platforms support redox-state sensing at the molecular level, reprinted with permission from Ref. [170], Springer. (**F**) FRET-based UCNP probes enable NIR-excited cancer biomarker detection with minimal background interference, reprinted with permission from Ref. [171], ACS.

A series of recent studies exemplify the versatility of UCNC-based platforms in achieving ultrasensitive and multiplexed SMD. Y. Luo et al. [167] used lanthanide-doped UCNs, as depicted in Figure 14A, employed in a ratiometric nanobeacon assay for miRNA detection, achieving a detection limit of 0.2 fM through dual-wavelength emission modulation. Similarly, a portable NIR-based immunosensor was designed by H. Tong et al. [167] using NaYF_4_:Yb,Er@NaYF_4_ UCNs for the detection of interleukin-6 (IL-6), achieving an LOD of 0.39 pg/mL with no cross-reactivity, as shown in Figure 14B. Moreover, Y. Zhang et al. [168] introduced a digital detection system using single UCN-labeled antibodies for the quantification of breast cancer markers in serum, with an LOD as low as 0.015 ng/mL, as depicted in Figure 14C. These studies showcase the translation of UCN-based SMD platforms toward ultrasensitive clinical assays and point-of-care testing.

Expanding beyond diagnostics, in 2024, as illustrated in Figure 14D, Shida J. F. [169] developed ligand-free UCNs with enhanced emission profiles by surface energy modulation, providing brighter signal intensities for low-abundance molecule detection in complex media. Then, Y. Luo et al. [170] demonstrated bright single-molecule upconversion electroluminescence in molecular junctions via spin-triplet mediated mechanisms, as presented in Figure 14E, showing an order-of-magnitude enhancement in emission intensity over previous approaches. Additionally, R. Lustig et al. [171] introduced a ligand–shell engineering strategy that enabled FRET efficiency tuning from 10% to over 65%, with a corresponding improvement in the signal-to-noise ratio exceeding 4.5 times in multiplexed bioassays, highlighting its value in precise and scalable SMD systems, as illustrated in Figure 14F.

As illustrated in Figure 14, these case studies collectively demonstrate the transformative potential of UCNCs in next-generation SMD platforms, delivering superior photostability, multiplexing, and background suppression relative to conventional optical probes. Despite these advancements, challenges such as limited quantum yields under low-power excitation, synthetic complexity, and potential cytotoxicity remain. To address these limitations, recent approaches have focused on energy migration engineering, surface passivation via inert shells, and ligand exchange for enhanced aqueous dispersibility and biocompatibility. Looking ahead, the integration of UCNCs with super-resolution microscopy, AI-assisted image reconstruction, and wearable or implantable biosensor systems is expected to unlock new frontiers in high-resolution, low-noise single-molecule diagnostics suitable for clinical and point-of-care applications [172,173].

### 3.5. MnO_2_ Nanosheets

Two-dimensional manganese dioxide (MnO_2_) nanosheets have emerged as a unique class of multifunctional nanomaterials with considerable promise for SMD. Owing to their high surface-to-volume ratios, variable oxidation states, and catalytic redox behavior, MnO_2_ nanosheets are well suited for signal amplification, quenching, and environmental responsiveness. These ultrathin nanosheets can act as oxidizing agents, peroxidase mimics, and fluorescence quenchers and can readily undergo reduction in biological or chemical microenvironments—features that allow them to function simultaneously as signal modulators and stimuli-responsive components [174]. Moreover, their ability to be integrated with DNA nanostructures, metal–organic frameworks (MOFs), and fluorescent probes enables the construction of sophisticated, tunable biosensing platforms that are both biocompatible and functionally rich. These properties collectively position MnO_2_ as a highly versatile material for next-generation biosensors operating in biologically relevant or field-deployable settings [175,176,177].

**Figure 15:**
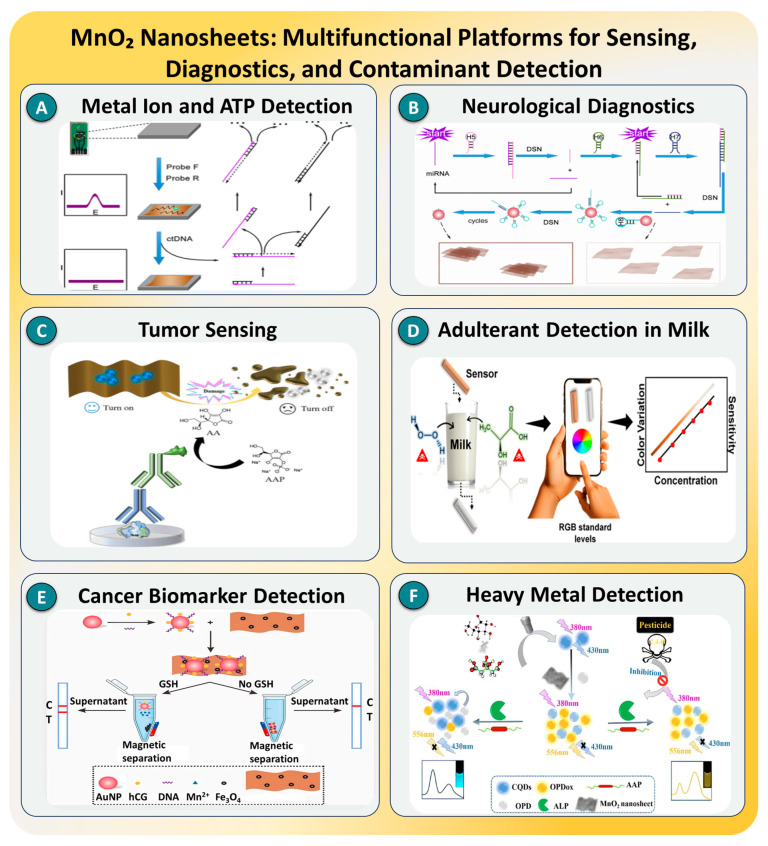
Illustrative overview of MnO_2_ nanosheet-based sensing strategies across diverse biomedical and environmental applications. (**A**) Electrochemical sensing of ATP and metal ions is achieved through MnO_2_-mediated signal modulation, reprinted with permission from Ref. [178], ACS. (**B**) DNA circuit-coupled MnO_2_ systems offer miRNA and neurobiomarker detection, reprinted with permission from Ref. [179], Elsevier. (**C**) Enzyme-triggered MnO_2_ nanosheets enable tumor microenvironment sensing, reprinted with permission from Ref. [180], Elsevier. (**D**) A smartphone-assisted colorimetric sensor detects milk adulterants through RGB-based color changes, reprinted with permission from Ref. [181] ACS. (**E**) Magnetic separation integrated with MnO_2_ nanosheets allows selective cancer biomarker recognition, reprinted with permission from Ref. [182], Elsevier. (**F**) A fluorescence-based platform identifies heavy metals and pesticides via MnO_2_-modulated emission quenching, reprinted with permission from Ref. [183], MDPI.

Recent innovations in MnO_2_ nanosheet-based nanoplatforms have unlocked significant potential in biosensing and therapeutics due to their redox activity, oxygen generation ability, and catalytic functionality. In this context, H. Chai [178] developed a DNAzyme–MnO_2_ platform for the dual detection of Cu^2+^ and ATP. The cleavage-induced release of fluorophore-labeled strands resulted in a detection limit of 0.13 nM for ATP, with strong selectivity in complex biological samples, as outlined in Figure 15A. In 2023, X. Ma [179] introduced a portable ratiometric fluorescence sensor based on MnO_2_ nanosheets and carbon quantum dots for dopamine sensing, as shown in Figure 15B. The method achieved a detection limit as low as 9.37 nM and demonstrated operational compatibility with human serum. Similarly, G. Zhang and team [180] used a fluorescence resonance energy transfer (FRET) system to sense glutathione in tumor environments using MnO_2_ nanosheet-induced quenching, as depicted in Figure 15C, where the detection limit reached 0.2 μM and excellent biocompatibility was observed in HeLa cells. Another key study by T. dos S. Pereira [181] presented a smartphone-integrated MnO_2_–zein film biosensor for detecting milk adulterants, as indicated in Figure 15D. This device utilized the peroxidase-like activity of MnO_2_ for colorimetric signal generation, achieving rapid detection in under 2 min with sensitivity in the μg/mL range.

Extending these capabilities, X. Gao [184] detailed the use of a mesoporous MnO_2_ nanocomposite for hydrogen peroxide (H_2_O_2_) detection, reaching a detection limit of 30 nM and proving valuable for reactive oxygen species quantification in oxidative stress models. Meanwhile, H. Yang et al. [182] integrated MnO_2_ nanosheets into a photothermal-augmented electrochemical sensor that targeted carcinoembryonic antigen, achieving ultralow detection down to 1.5 fg/mL, as shown in Figure 15E. In 2024, Y. Guo et al. [183] developed a dual-mode system combining MnO_2_ and silver nanoclusters (AgNCs) for the simultaneous fluorescence and SERS detection of Hg^2+^ ions, with a limit of 0.5 nM, as illustrated in Figure 15F. Most recently, Y. Zheng et al. [185] demonstrated a multifunctional platform integrating DNA tetrahedrons, MnO_2_ nanosheets, and photosensitizers for synergistic gene–photodynamic therapy in cancer. The system alleviated tumor hypoxia-activated DNAzymes with Mn^2+^ and improved PDT’s efficacy under near-infrared light, achieving significant tumor suppression in vivo.

As illustrated in Figure 15, these case studies highlight the role of MnO_2_ nanosheets in expanding the operational boundaries of single-molecule biosensors. Their intrinsic catalytic and redox-switchable properties enable dynamic, stimuli-responsive sensing under physiological conditions. Moreover, their integration into modular platforms, ranging from smartphone interfaces to gene-activated nanostructures, demonstrates their versatility across clinical, environmental, and theranostic applications. However, limitations persist, including potential Mn^2+^ cytotoxicity, nanosheet aggregation, and variable reduction kinetics in complex biological matrices [186]. To address these concerns, future research is focusing on structural doping, hybridization with biopolymers or inert shells, and the use of microfluidic confinement or AI-assisted calibration to ensure stability, selectivity, and reproducibility. With continued advancement, MnO_2_ nanosheets are poised to play a central role in the development of smart, multifunctional SMD platforms with real-time feedback, therapeutic integration, and translational potential [187,188].

### 3.6. Magnetic Nanoparticles

Magnetic nanoparticles (MNPs), particularly those based on iron oxide, exhibiting superparamagnetic properties, have become integral to SMD systems due to their unique abilities to facilitate magnetic enrichment, dynamic manipulation, and noncontact signal modulation. These nanoscale materials, typically composed of Fe_3_O_4_ or γ-Fe_2_O_3_, possess large surface-to-volume ratios, high magnetic responsiveness, and excellent biocompatibility. Their surfaces can be readily functionalized with antibodies, aptamers, or DNA probes, allowing for highly selective target recognition [189,190]. A key advantage of MNPs lies in their controllability via external magnetic fields, which enables precise spatial localization, rapid separation, and the temporal modulation of molecular events. These features support their integration into various detection modalities, including magnetic relaxation switching, magneto-optical transduction, and electrochemical impedance platforms, thereby enhancing the sensitivity, automation, and miniaturization potential in SMD workflows.

**Figure 16:**
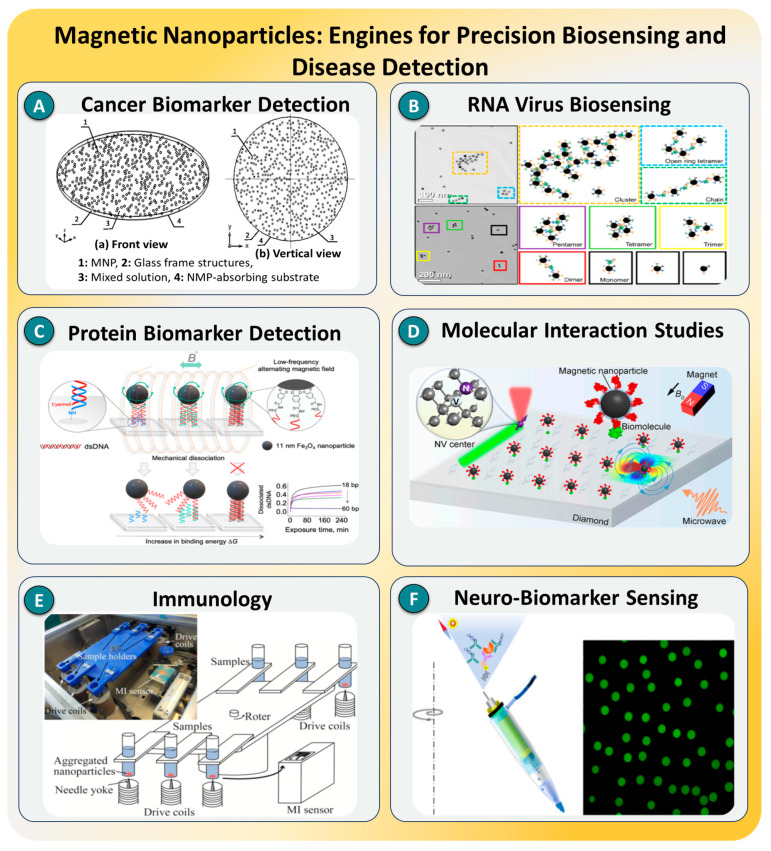
This figure illustrates diverse magnetic nanoparticle (MNP)-based strategies for SMD across biomedical domains. (**A**) Magnetically assembled SERS substrates for ultrasensitive cancer biomarker detection, reprinted with permission from Ref. [191], MDPI. (**B**) Magnetoplasmonic aggregation for RNA virus biosensing, reprinted with permission from Ref. [192], ACS. (**C**) Digital RCA assay for protein biomarker quantification using magnetic dissociation, reprinted with permission from Ref. [193], ACS. (**D**) NV center-based quantum sensing for molecular interaction studies, reprinted with permission from Ref. [194], ACS. (**E**) Barcode-tagged MNPs for multiplex cytokine profiling, reprinted with permission from Ref. [195], AIP. (**F**) Centrifugal droplet immuno-PCR for neurodegenerative biomarker detection, reprinted with permission from Ref. [196], ACS.

Magnetic nanoparticles (MNPs) have become integral to SMD technologies due to their magnetic responsiveness, large surface areas, and adaptability to diverse detection modalities. To this end, L. Wang [191] described magnetically assembled SERS substrates, which enabled the ultrasensitive detection of cancer biomarkers down to 10^−14^, as represented in Figure 16A. Thereafter, M. K. Wu et al. [192] demonstrated a dual-mode biosensor integrating Fe_3_O_4_@Au nanoparticles for viral RNA detection at ~100 aM by combining magnetic pre-concentration with plasmonic enhancement, as illustrated in Figure 16B. Similarly, in A. A. Nikitin et al. [193], a rolling circle amplification (RCA)-based platform coupled with MNPs achieved protein detection at ~50 aM, as shown in Figure 16C, while S. Chen et al. [194] tracked the Brownian motion of MNPs using optical tweezers for the real-time monitoring of single-molecule interactions, as shown in Figure 16D. In 2024, S. Yabukami [195] employed barcode-tagged MNPs for simultaneous cytokine quantification in serum with a sub-10 pg/mL resolution, as illustrated in Figure 16E. In the same year, A. Camarca et al. [196] presented a centrifugal droplet immuno-PCR strategy using Au-coated MNPs for α-synuclein detection, as indicated in Figure 16F, achieving ~50 aM sensitivity in buffer and ~170 aM in serum.

As illustrated in Figure 16, these studies collectively demonstrate the multifaceted utility of magnetic nanoparticles in SMD platforms, particularly in achieving high-efficiency enrichment, contactless actuation, and signal enhancement. The ability to precisely manipulate magnetic fields provides unparalleled control over molecular recognition and readout, making MNPs ideal candidates for integration with point-of-care, wearable, and automated diagnostic systems [197,198]. Nonetheless, challenges such as magnetic aggregation, limited colloidal stability under physiological conditions, and nonspecific interactions remain. Current solutions include surface PEGylation, charge shielding, and incorporation into hybrid nanostructures with plasmonic or fluorescent elements. Looking ahead, the fusion of MNPs with AI-assisted signal processing, digital microfluidics, and quantum sensing technologies is anticipated to further elevate the performance of magnetic nanoparticle-based SMD toward real-time, ultrasensitive, and field-deployable biosensing [199].

## 4. Different Applications of SMD

**Figure 17:**
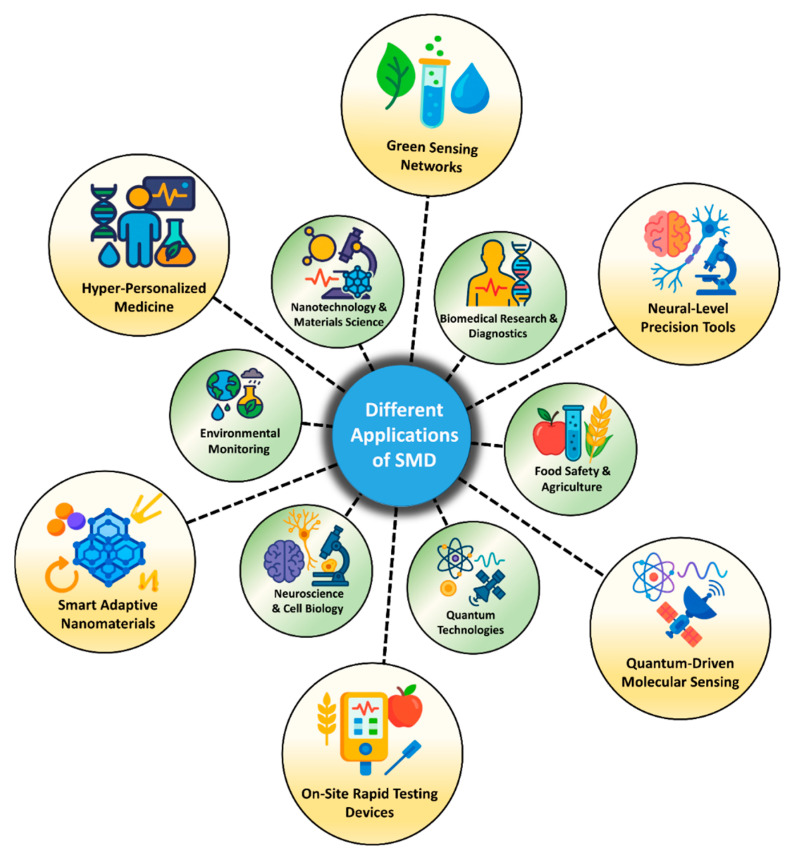
Schematic representation of the diverse and evolving application domains of SMD, spanning biomedical diagnostics, environmental monitoring, food safety, neuroscience, nanotechnology, and quantum technologies. The figure also highlights emerging frontiers such as hyper-personalized medicine, quantum-driven molecular sensing, and adaptive green sensing networks. By enabling ultrasensitive, label-free, and real-time molecular interrogation, SMD is poised to revolutionize both fundamental research and translational technologies.

The capability to detect and analyze individual molecules has led to significant breakthroughs across a broad spectrum of scientific and technological domains. By surpassing the limitations of ensemble averaging, SMD offers unparalleled sensitivity and resolutions, enabling the direct observation of molecular heterogeneity, rare biomolecular events, and transient intermediate states that are often obscured in bulk measurements. This level of precision is particularly valuable in complex biological and chemical systems, where individual molecular interactions dictate system behavior [31,200]. Figure 17 provides a schematic overview of the diverse and expanding application domains of SMD, including both established and emerging fields. As a result, SMD has become a pivotal analytical tool in a wide array of applications, including early-stage disease diagnostics, real-time environmental pollutant monitoring, food quality assurance, nanomaterial characterization, cellular and neurobiological investigations, and quantum-enabled metrology. To provide a holistic understanding of its utility across fields, the diverse applications of SMD are benchmarked in Table 2, with detailed discussions provided in the subsequent sections.

### 4.1. Biomedical Research and Diagnostics

SMD is becoming a gamechanger in the field of biomedicine, especially for applications like early disease detection, understanding disease mechanisms, and designing new drugs. What makes SMD so powerful is its ability to detect and study individual molecules—something that traditional methods, which average signals from large groups of molecules, simply cannot achieve. In biomarker detection, for instance, techniques like surface plasmon resonance (SPR) and fluorescence-based sensing have shown remarkable sensitivity. They can detect disease-related molecules such as HER2 and C-reactive protein at extremely low levels, sometimes even at attomolar concentrations, in body fluids like blood serum or saliva [201]. This makes it possible to identify diseases, including certain cancers, long before symptoms appear, providing opportunities for early intervention and better treatment outcomes.

SMD is also proving highly useful in drug discovery and development. By watching how single drug molecules interact with their targets in real time, researchers can understand how tightly they bind, how rapidly they work, and whether they cause unwanted side effects. Mechanochemical sensors and FET-based biosensors are especially useful in high-throughput drug screening, helping to speed up the discovery of effective and safe drug candidates [202]. In the field of genomics and proteomics, SMD technologies like nanopore-based transistors and recognition tunneling are making it possible to detect DNA, RNA, and proteins without needing labels or amplification [203]. These tools offer extremely high resolutions down to a single base pair in DNA or a single amino acid in proteins, which is vital for personalized medicine. This level of detail helps to identify genetic mutations or protein misfolding events that can lead to diseases, enabling more precise and tailored treatments for individual patients. Overall, SMD is not just enhancing our ability to see the molecular world in detail; it is opening new pathways for the diagnosis, treatment, and understanding of diseases at the most fundamental level.

### 4.2. Environmental Monitoring

SMD technologies have proven to be powerful analytical tools for assessing environmental quality through the ultrasensitive detection of pollutants. Their ability to detect trace analytes in complex media enables the real-time monitoring of contaminants across air, water, and soil systems. In pollutant detection, techniques such as break junction sensing and mechanochemical transduction have been effectively used to identify heavy metals like mercury (Hg^2+^) through molecular probes such as fluorescein–phenylalaninol conjugates [204]. These platforms also support the detection of volatile organic compounds and pesticide residues at sub-nanomolar levels, thereby offering a rapid and reliable approach to monitoring water and soil contamination [205]. SMD systems have also been extended to air quality and microplastic monitoring. Optical and Raman scattering-based SMD devices enable the in situ detection of airborne nanoparticles and polymer debris, facilitating the tracking of particulate pollution and microplastic infiltration in the atmosphere. These tools help to generate spatially resolved contamination maps, crucial for regulatory assessment and ecological intervention.

### 4.3. Food Safety and Agriculture

SMD technologies are increasingly being integrated into food safety and agricultural diagnostics due to their noninvasive, label-free, and high-sensitivity nature. These properties allow for the early detection of contaminants and pathogens, even in heterogeneous or minimally processed samples. In food contaminant detection, optical microfiber sensors and surface plasmon resonance (SPR)-based SMD platforms have been successfully utilized to detect trace allergens (e.g., hazelnut protein in dairy alternatives), bacterial toxins, and spoilage-related volatile compounds [206,207]. These systems offer real-time, on-site diagnostics that ensure food quality and consumer safety. For crop disease diagnosis, SMD methods such as fluorescence-tagged molecular probes and FET-based biosensors have demonstrated promising results in the early detection of plant pathogens and microbial infections [208]. These tools help in identifying disease onset at the molecular level, enabling proactive crop management and minimizing agricultural losses.

### 4.4. Nanotechnology and Materials Science

SMD plays a pivotal role in nanotechnology by enabling the molecular-level analysis of nanomaterials, aiding in the design and functional optimization of nanoscale systems. It facilitates the precise investigation of material behavior, interfaces, and interactions. In nanoparticle characterization, fluorescence-based and Raman-assisted SMD methods allow the accurate determination of the size distribution, surface charge, optical emission properties, and functionalization efficiency of nanomaterials [209]. These techniques are essential for applications in targeted drug delivery, diagnostics, and catalysis. Furthermore, in surface chemistry and molecular assembly, single-molecule tunneling spectroscopy and other SMD modalities provide critical insights into self-assembled monolayers, ligand exchange dynamics, and catalytic mechanisms on 2D materials such as graphene, MoS_2_, and MnO_2_ nanosheets [210,211]. This knowledge is vital in advancing material performance in electronics, energy storage, and biosensing.

### 4.5. Neuroscience and Cellular Biology

The inherently stochastic and highly dynamic environments of neuronal and cellular systems require detection tools with exceptional temporal resolutions and sensitivity capabilities, as offered by SMD. In neuronal signaling and synaptic function, fluorescence-based SMD platforms have been instrumental in tracking neurotransmitter release, vesicle docking, and calcium ion flux at individual synapses. These measurements are essential for deciphering the molecular basis of neural plasticity, learning, and memory [209]. SMD also supports single-cell molecular profiling, where optical microfiber probes and transistor-based nanosensors have been used to monitor gene expression levels, protein folding pathways, and intracellular trafficking within individual live cells [212]. These tools are invaluable for cell type classification, disease modeling, and therapeutic response prediction at a single-cell resolution.

### 4.6. Quantum Technologies and Metrology

SMD technologies are intersecting increasingly with the field of quantum science, where they serve as key enablers of ultrasensitive measurement and quantum-scale sensing. In quantum-enhanced sensing, tunneling-based SMD platforms such as scanning tunneling microscopy (STM) and break junctions facilitate the detection of discrete charge carriers, single electrons, and photons [104,213]. These capabilities underpin developments in quantum cryptography, metrology, and low-noise amplification systems. SMD has also contributed significantly to high-resolution imaging and spectroscopy, enabling the detection of molecular transitions and exciton dynamics beyond the classical diffraction limit. These advancements support both fundamental research in quantum optics and practical applications in materials science, where atomic-scale precision is critical.

Finally, the breadth of SMD applications underscores its transformative impact across the biomedical, environmental, agricultural, and emerging quantum domains. Each field leverages the core strengths of SMD—ultrasensitivity, label-free operation, and real-time single-event analysis—to solve unique challenges, ranging from early disease diagnosis to nanomaterial characterization and environmental toxin monitoring. Despite domain-specific adaptations, common bottlenecks persist, including assay standardization, device robustness, and data complexity in uncontrolled settings. Moving forward, the convergence of SMD with portable diagnostics, AI-enhanced analytics, and cloud-integrated sensing frameworks is expected to drive global adoption. As the field matures, SMD is poised not only to advance fundamental science but also to become a mainstay technology for addressing real-world health, safety, and sustainability challenges. The following Table 2 provides a comparative overview of SMD applications across key domains, highlighting the detection limits, label-free operation, platform types, and representative sensing techniques.

**Table 2:** Applications and key roles of SMD.

Application Domain	Detection Limit	Label-Free	Platform Type	Key Role of SMD	Representative Techniques	Ref.
Biomedical Research and Diagnostics	Attomolar to femtomolar	Yes	FET chips, nanopores, microfluidics	Early disease diagnosis, drug discovery, real-time biomolecular interaction studies	SPR, fluorescence sensing, FET biosensors, nanopore sensing	[214,215]
Environmental Monitoring	Sub-nanomolar to nanomolar	Yes	Break junctions, field probes	Trace detection of pollutants (e.g., heavy metals, VOCs), microplastic mapping	Break junction sensing, mechanochemical transduction, Raman scattering	[216]
Food Safety and Agriculture	Nanomolar	Yes	SPR chips, optical fibers	Noninvasive detection of allergens, pathogens, and spoilage indicators	SPR, optical microfiber sensors, FET biosensors	[217,218]
Nanotechnology and Materials Science	Single-molecule resolution	Depends on probe	Scanning probes, 2D platforms	Molecular-level characterization of nanomaterials, surface chemistry studies	Fluorescence imaging, Raman spectroscopy, tunneling spectroscopy	[219,220]
Neuroscience and Cellular Biology	Single-molecule, single-vesicle	Yes	Microfibers, nanoelectrodes	Real-time monitoring of neurotransmitter dynamics, single-cell analysis	Fluorescence-based probes, optical microfibers, nanoelectrodes	[221,222]

## 5. Challenges and Future Perspectives

**Figure 18:**
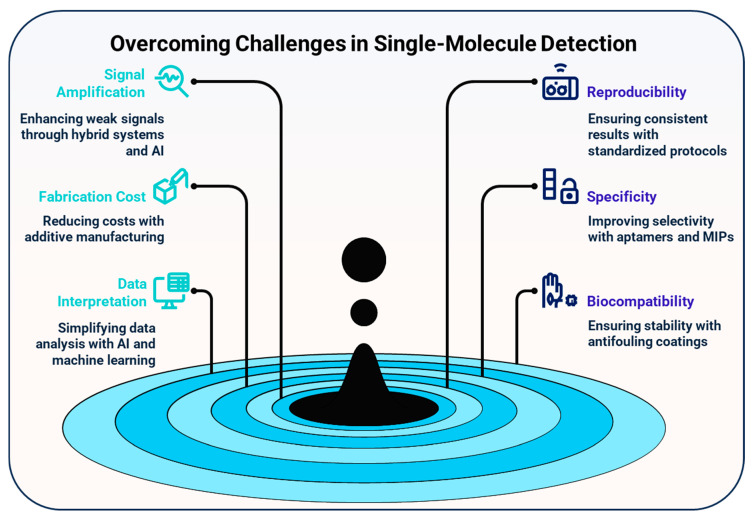
Schematic representation of key challenges and proposed solutions in SMD systems.

Despite substantial progress in the development of SMD technologies, their transition from laboratory prototypes to robust, deployable platforms remains constrained by several critical challenges. These limitations are multifaceted, encompassing material-level inconsistencies, transduction inefficiencies, fabrication complexities, and interpretational bottlenecks. Addressing these barriers is imperative to enhance the reliability, reproducibility, and scalability of SMD systems across biomedical, environmental, and industrial domains. This section systematically delineates the prevailing challenges, critiques methodological gaps in current research, and proposes forward-looking strategies that leverage recent advances in materials science, data science, and device engineering (Figure 18).

### 5.1. Signal Amplification and Sensitivity Limitations

A fundamental challenge in SMD lies in the inherently weak signal outputs generated by individual molecular events. Optical techniques such as fluorescence and Raman scattering often suffer from issues such as photobleaching, spectral crosstalk, and background autofluorescence, particularly in biological environments [219]. Likewise, electronic and mechanical approaches frequently encounter low signal-to-noise ratios (SNRs), especially when detecting transient or low-abundance targets. These limitations impair the ability to detect rare molecular interactions and reduce the confidence in quantitative analysis [223].

From a methodological perspective, most existing approaches rely on conventional signal enhancement techniques such as plasmonic nanoparticles, resonant cavities, or chemical amplification yet often neglect long-term stability and cross-sample variability. There is a pressing need to develop hybrid transduction systems that integrate multiple physical modalities (e.g., electro-optical, mechano-electrical) to improve the sensitivity and noise rejection. Additionally, incorporating AI-based signal reconstruction models (e.g., convolutional autoencoders or transformer networks) can significantly enhance the detection fidelity by distinguishing molecular signals from environmental or instrumental noise in real time [224,225].

### 5.2. Reproducibility and Material Inconsistency

Reproducibility remains a critical bottleneck in the development of SMD platforms, particularly those employing nanomaterials such as graphene, quantum dots, carbon nanotubes, and MnO_2_ nanosheets. The physicochemical properties of these materials, such as the surface charge, functionalization density, and morphology, often exhibit batch-to-batch variability, which directly influences sensor performance. Moreover, inconsistencies in surface functionalization chemistry lead to nonuniform molecular recognition and signal transduction across sensor units [225].

The current literature frequently lacks standardized protocols for synthesis, functionalization, and performance benchmarking, thereby limiting cross-study comparison and clinical translation. To mitigate these limitations, future research should prioritize the implementation of reproducible fabrication techniques (e.g., microfluidic-assisted synthesis, template-directed growth) combined with robust surface passivation strategies such as PEGylation or zwitterionic coatings. Furthermore, the integration of digital twin frameworks—AI-powered virtual replicas of physical sensors—can facilitate real-time calibration, predictive modeling, and consistency assessment across distributed sensor networks [226,227].

### 5.3. High Fabrication Costs and Lack of Scalability

Most state-of-the-art SMD systems are fabricated using resource-intensive techniques such as electron beam lithography, focused ion beam milling, and nanoimprinting, which are unsuitable for large-scale production [228]. These processes demand cleanroom infrastructure, specialized expertise, and extended production times, significantly increasing the cost and limiting accessibility in low-resource settings. Despite the widespread recognition of this limitation, there is a paucity of research focused on economic feasibility and manufacturing scalability. A promising direction involves the adoption of additive manufacturing techniques such as inkjet or aerosol printing on flexible substrates, enabling the cost-effective, roll-to-roll fabrication of nanostructured sensors [229]. Concurrently, integrating these sensors with portable analytical platforms (e.g., smartphone-based imaging systems, low-power signal processors) can democratize the deployment of SMD technologies in decentralized clinical diagnostics, environmental monitoring, and agricultural testing [230,231].

### 5.4. Specificity and Selectivity in Complex Matrices

The practical deployment of SMD platforms is often constrained by limited specificity and selectivity in complex matrices (blood, saliva, environmental extracts), where nonspecific binding, matrix-induced quenching, and biomolecular fouling can cause false results and degrade accuracy. A key gap is the limited evaluation under physiologically or environmentally relevant conditions. To address this, highly selective recognition interfaces, aptamers, MIPs, and engineered peptide receptors are essential, while multi-modal readouts (e.g., SPR with electrochemical impedance spectroscopy) help to validate signals and mitigate cross-reactivity. Notably, several real-sample studies highlight these effects: an electrochemical Ti_3_C_2_T_x_-MXene/4-APBA sensor detected dopamine in human serum, where protein fouling slightly increased the LOD from 1.3 nM to 1.9 nM but preserved the linearity and precision [232]; an Au–Au nanorod SERS system quantified thiram on cucumber skin, overcoming spectral overlap through ML deconvolution with ~7% RSD [233]; an SPR assay measured therapeutic and anti-drug antibodies in undiluted serum, showing minor baseline drift yet maintaining therapeutic-range linearity [234]; and a nanopore analysis of urine profiled metabolites under high ionic interference, yielding LC-MS/MS-matched fingerprints without loss of accuracy [235]. Together with ensemble and deep-kernel ML for robust signal classification, these advances enhance the reliability of SMD’s performance in heterogeneous samples.

### 5.5. Data Interpretation and Computational Complexity

The high-dimensional, real-time data generated by modern SMD systems pose considerable challenges for storage, analysis, and interpretation. Traditional statistical methods are often inadequate in capturing the stochastic, nonlinear, and sparse nature of single-molecule events. Furthermore, human-led data annotation is labor-intensive, subjective, and poorly scalable [236]. To overcome these methodological constraints, the integration of AI and machine learning into the SMD data processing pipeline is essential. Time-series modeling techniques such as LSTM networks, hidden Markov models (HMMs), and Bayesian inference can be leveraged for event classification, binding kinetics extraction, and noise filtering. Real-time analysis can be enabled through edge AI frameworks, wherein compact ML models operate directly on-device, facilitating rapid decision making without cloud dependency. In clinical applications, explainable AI (XAI) methods should be prioritized to ensure transparency, traceability, and regulatory compliance [237,238].

### 5.6. Biocompatibility and In Vivo Stability

For in vivo and implantable SMD systems, long-term biocompatibility and structural stability are essential. In physiological environments, nonspecific protein adsorption, immune activation, and biofouling often reduce sensor performance and limit its lifespan [239]. Among the functional materials discussed in Section 3, graphene and carbon nanotubes provide superior conductivity and surface areas but may trigger oxidative stress or inflammation unless modified with PEG or chitosan coatings. Quantum dots offer bright, stable emission yet elicit concerns over heavy metal toxicity, prompting the development of cadmium-free alternatives. Gold nanoparticles are generally inert and cytocompatible, whereas silver or certain polymers may cause oxidative damage during extended exposure. Upconversion nanocrystals show promising optical performance with moderate biocompatibility, although rare earth components require further toxicological study. MnO_2_ nanosheets contribute catalytic activity but can disturb the cellular redox balance at high concentrations, while iron oxide magnetic nanoparticles demonstrate excellent hemocompatibility and remain the most established for biomedical use [240].

To improve the in vivo stability, antifouling surface chemistries, such as mussel-inspired dopamine coatings, zwitterionic hydrogels, and lipid-bilayer modifications, are being increasingly adopted. Dynamic surface renewal strategies, including electrochemical regeneration and photonic cleaning, help to sustain signal integrity under physiological conditions [136]. Overall, noble metal, MXene, and iron oxide interfaces show strong potential for long-term biomedical deployment, whereas unmodified carbon nanostructures and heavy metal-based quantum dots require further toxicological refinement before safe clinical translation [241].

## 6. Conclusions and Future Directions

SMD has reformed analytical science by enabling the real-time, label-free detection of molecular events at unparalleled sensitivity. Through the integration of advanced transduction mechanisms and novel materials like graphene, quantum dots, MnO_2_ nanosheets, and upconversion nanocrystals, SMD platforms now support detection limits down to the zeptomolar range. These capabilities are driving progress in biomedical diagnostics, environmental monitoring, food safety, and quantum metrology. However, the widespread translation of SMD technologies into real-world settings is limited by challenges such as surface fouling, signal reproducibility, device scalability, and computational complexity.

To overcome these barriers, future research must focus on hybrid sensing architectures, AI-driven signal interpretation, bioinspired surface coatings, and standardized manufacturing protocols. Emerging trends include the development of wearable and implantable sensors, quantum-enhanced platforms, and sustainable, roll-to-roll fabrication methods for scalable deployment. Machine learning tools will play a vital role in distinguishing true molecular signals from noise, enabling more robust and interpretable data outputs. By aligning innovations in transduction, materials, and data science, the next generation of SMD systems is poised to move beyond the laboratory into everyday healthcare, environmental safety, and precision agriculture. This convergence promises to elevate SMD as a cornerstone of personalized and predictive diagnostics in the coming decade.

## Data Availability

No new data were created or analyzed in this study.
